# Genotyping of European *Toxoplasma gondii* strains by a new high-resolution next-generation sequencing-based method

**DOI:** 10.1007/s10096-023-04721-7

**Published:** 2023-12-15

**Authors:** M. Joeres, P. Maksimov, D. Höper, S. Calvelage, R. Calero-Bernal, M. Fernández-Escobar, B. Koudela, R. Blaga, M. Globokar Vrhovec, K. Stollberg, N. Bier, S. Sotiraki, J. Sroka, W. Piotrowska, P. Kodym, W. Basso, F. J. Conraths, A. Mercier, L. Galal, M. L. Dardé, A. Balea, F. Spano, C. Schulze, M. Peters, N. Scuda, A. Lundén, R. K. Davidson, R. Terland, H. Waap, E. de Bruin, P. Vatta, S. Caccio, L. M. Ortega-Mora, P. Jokelainen, G. Schares

**Affiliations:** 1 Friedrich-Loeffler-Institut, Federal Research Institute for Animal Health, Institute of Epidemiology, Greifswald - Insel Riems, Germany; 2 Friedrich-Loeffler-Institut, Federal Research Institute for Animal Health, Institute of Diagnostic Virology, Greifswald - Insel Riems, Germany; 3 SALUVET, Animal Health Department, Faculty of Veterinary Sciences, Complutense University of Madrid, Madrid, Spain; 4 Central European Institute of Technology (CEITEC), University of Veterinary Sciences Brno, Brno, Czech Republic; 5 Faculty of Veterinary Medicine, University of Veterinary Sciences Brno, Brno, Czech Republic; 6 Anses, INRAE, Ecole Nationale Vétérinaire d’Alfort, Laboratoire de Santé Animale, BIPAR, Maisons-Alfort, France; 7 University of Agricultural Sciences and Veterinary Medicine, Cluj-Napoca, Romania; 8 IDEXX Laboratories, Kornwestheim, Germany; 9 German Federal Institute for Risk Assessment, Department for Biological Safety, Berlin, Germany; 10 Veterinary Research Institute, Hellenic Agricultural Organisation-DIMITRA, Thessaloniki, Greece; 11 Department of Parasitology and Invasive Diseases, National Veterinary Research Institute, Pulawy, Poland; 12 Centre of Epidemiology and Microbiology, National Institute of Public Health, Prague, Czech Republic; 13 Institute of Parasitology, Vetsuisse Faculty, University of Bern, Bern, Switzerland; 14 Inserm U1094, IRD U270, Univ. Limoges, CHU Limoges, EpiMaCT - Epidemiology of chronic diseases in tropical zone, Institute of Epidemiology and Tropical Neurology, OmegaHealth, Limoges, France; 15 Centre National de Référence (CNR) Toxoplasmose Centre Hospitalier-Universitaire Dupuytren, Limoges, France; 16 University of Agricultural Sciences and Veterinary Medicine Cluj-Napoca, Faculty of Veterinary Medicine, Department of Parasitology and Parasitic Diseases, Cluj-Napoca, Romania; 17 Italian National Institute of Health, Rome, Italy; 18 Landeslabor Berlin-Brandenburg, Frankfurt (Oder), Germany; 19 Chemisches und Veterinäruntersuchungsamt Westfalen, Standort Arnsberg, Arnsberg, Germany; 20 Bavarian Health and Food Safety Authority, Erlangen, Germany; 21 Department of Microbiology, National Veterinary Institute, Uppsala, Sweden; 22 Department of Animal Health, Welfare and Food Safety, Norwegian Veterinary Institute, Tromsø, Norway; 23 Department of Analysis and Diagnostics, Norwegian Veterinary Institute, Ås, Norway; 24 Parasitology Laboratory, Instituto Nacional de Investigação Agrária e Veterinária, Oeiras, Portugal; 25 Dutch Wildlife Health Centre, Pathology Division, Department of Pathobiology, Faculty of Veterinary Medicine, University of Utrecht, Utrecht, The Netherlands; 26 Infectious Disease Preparedness, Statens Serum Institut, Copenhagen, Denmark

**Keywords:** Typing, Discriminatory power, Intra-genotype variability, Highly polymorphic regions, Multilocus sequence typing, Toxoplasmosis

## Abstract

**Purpose:**

A new high-resolution next-generation sequencing (NGS)-based method was established to type closely related European type II *Toxoplasma gondii* strains.

**Methods:**

*T. gondii* field isolates were collected from different parts of Europe and assessed by whole genome sequencing (WGS). In comparison to ME49 (a type II reference strain), highly polymorphic regions (HPRs) were identified, showing a considerable number of single nucleotide polymorphisms (SNPs). After confirmation by Sanger sequencing, 18 HPRs were used to design a primer panel for multiplex PCR to establish a multilocus Ion AmpliSeq typing method. *Toxoplasma gondii* isolates and *T. gondii* present in clinical samples were typed with the new method. The sensitivity of the method was tested with serially diluted reference DNA samples.

**Results:**

Among type II specimens, the method could differentiate the same number of haplotypes as the reference standard, microsatellite (MS) typing. Passages of the same isolates and specimens originating from abortion outbreaks were identified as identical. In addition, seven different genotypes, two atypical and two recombinant specimens were clearly distinguished from each other by the method. Furthermore, almost all SNPs detected by the Ion AmpliSeq method corresponded to those expected based on WGS. By testing serially diluted DNA samples, the method exhibited a similar analytical sensitivity as MS typing.

**Conclusion:**

The new method can distinguish different *T. gondii* genotypes and detect intra-genotype variability among European type II *T. gondii* strains. Furthermore, with WGS data additional target regions can be added to the method to potentially increase typing resolution.

**Supplementary Information:**

The online version contains supplementary material available at 10.1007/s10096-023-04721-7.

## Introduction

*Toxoplasma gondii* is a zoonotic protozoon that infects a large variety of warm-blooded species and can cause clinical disease in animals and humans. Felids are the definitive hosts of this parasite with sexual reproduction stage occurring only in their intestines [1–5]. In a European study, *T. gondii* was ranked second out of 24 important foodborne parasites [6]. At a global level, *T. gondii* has a complex population structure [7]. While clonal lineages dominate many regions [8], the *T. gondii* population is diverse in other parts of the world, like South America [7, 9].

A frequently used genotyping method for *T. gondii* assesses up to 15 microsatellite (MS) markers located in 11 different chromosomes. This method includes eight lineage typing and seven fingerprinting markers, the latter being more polymorphic and thus able to detect variability within archetypal (type I, II or III) and non-archetypal lineages [10]. MS typing represents the current reference standard for genotyping and fingerprinting. Harmonized guidelines were recently established to reach consistency between different laboratories [11]. Since data analysis cannot be completely automated, interpretation of MS typing results is affected by user experience and software for data analyses [11].

Another commonly used method to type *T. gondii* is PCR-restriction fragment length polymorphism (PCR-RFLP). This method involves up to 11 markers, distributed over eight chromosomes and the apicoplast genome [12]. It can differentiate genotypes, but cannot detect intra-genotype variability.

Multilocus sequence typing (MLST) of *T. gondii* [13, 14] targets specific regions in the parasite genome and was in past studies based on Sanger sequencing*.* If the amount of *T. gondii* DNA is not limited, MLST is an efficient technique due to its high typing resolution [15], because it displays the whole variability of a sequenced region. Due to the broad application of next generation sequencing (NGS) and the advantages compared to Sanger sequencing, NGS should replace Sanger sequencing for MLST of *T. gondii*. While only a single DNA fragment can be sequenced at a time with Sanger sequencing, millions of fragments are sequenced simultaneously per run with NGS. This allows to multiplex several highly polymorphic regions (HPRs) and different samples in a single sequencing run. Compared to Sanger sequencing, NGS also has higher sensitivity in detecting rare variants due to deep sequencing.

Furthermore, whole genome sequencing (WGS) provides the most detailed information about genetic variability. However, WGS requires highly concentrated DNA and may not be suitable for laboratories with more limited resources [16]. In addition, WGS of *T. gondii* is a bioinformatically challenging task due to the size of its genome, approximately 65 Mb [9].

*T. gondii* type II is the predominant clonal genotype in Europe, but MS typing and WGS analysis revealed genetic variability within this lineage [8, 9, 17, 18]. A high resolution MLST method, which is easy to interpret, is needed to improve our understanding of *T. gondii* transmission pathways, to analyze outbreaks and trace infection sources in a setting such as Europe. We aimed to establish a NGS-based typing method with a high typing resolution among closely related type II strains that allows for automated and standardized data analysis. This new typing method should help to better understand the molecular epidemiology and transmission pathways of *T. gondii* in Europe.

## Material and methods

### Collection of specimens, DNA extraction and quantification

**Fig. 1:**
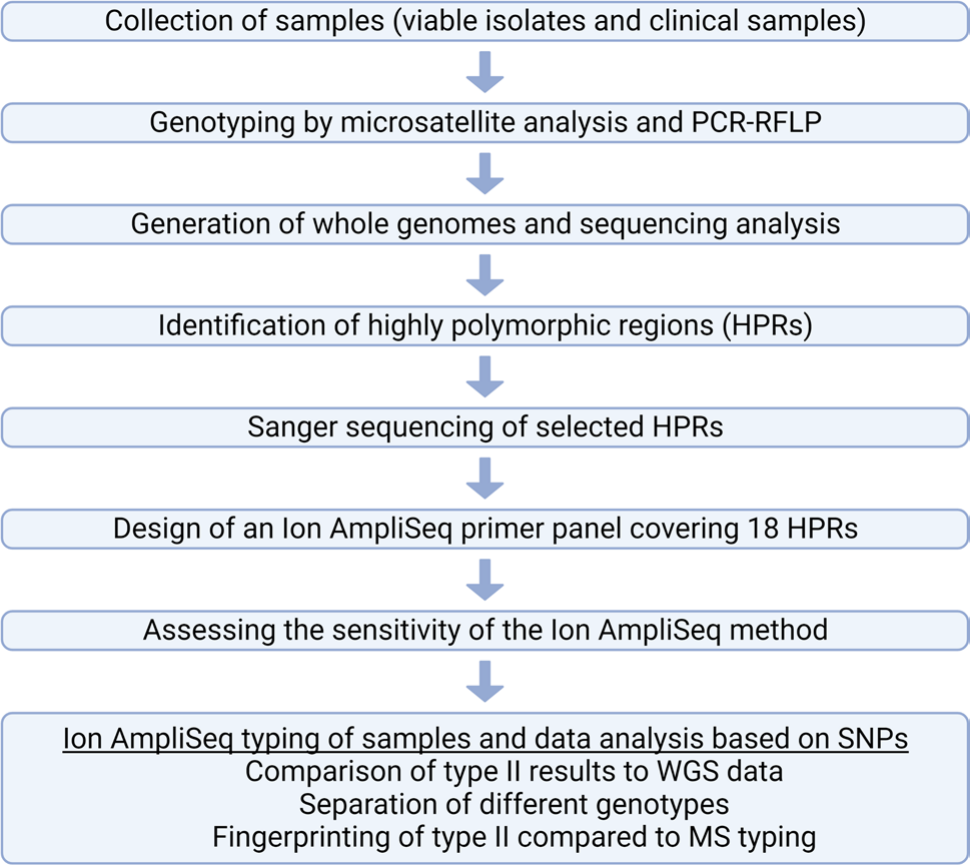
Workflow of the establishment of the Ion AmpliSeq method. Created with BioRender.com

**Fig. 2:**
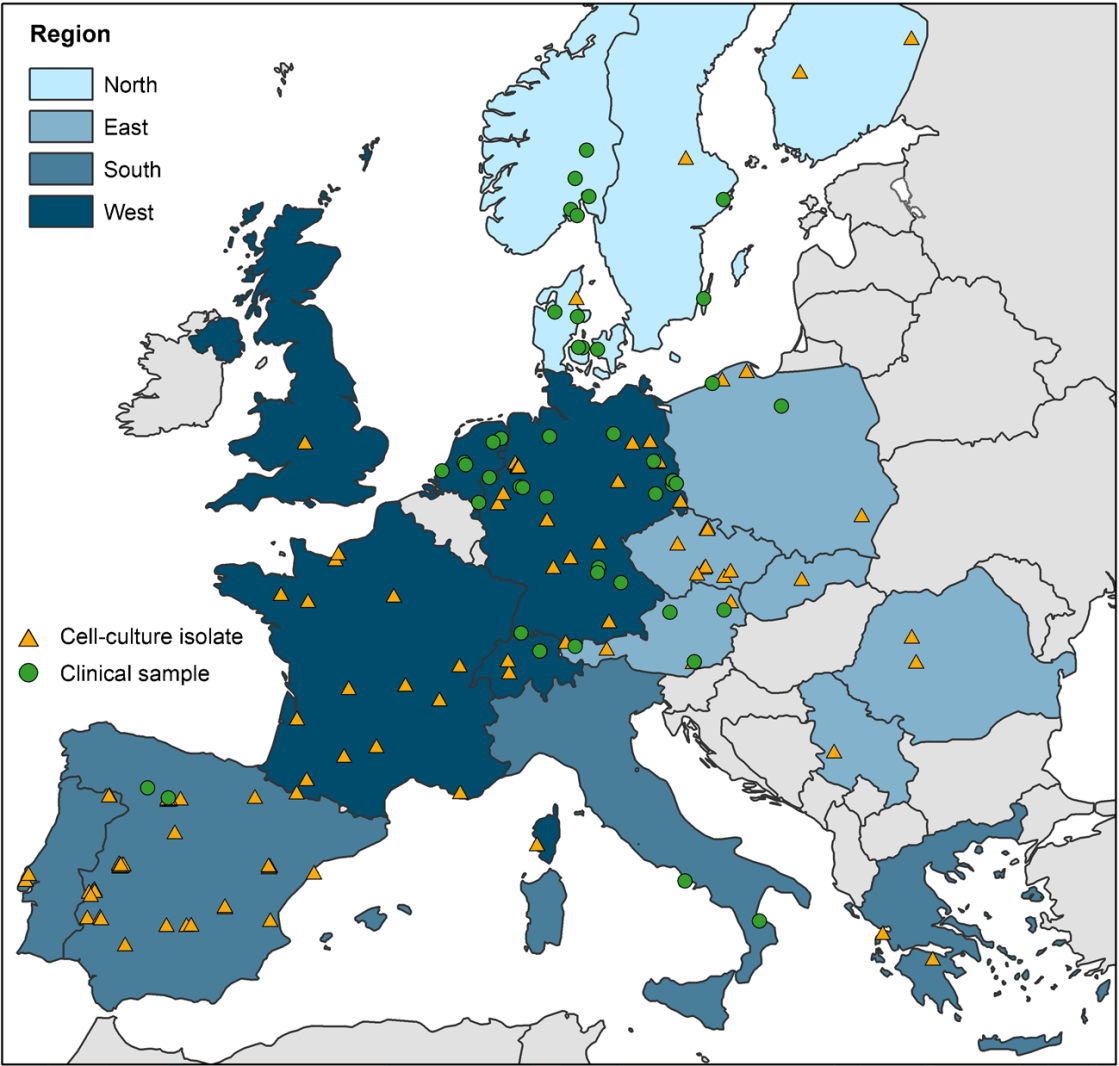
Geographic origin of 110 European *T. gondii* isolates and 47 clinical samples, which were genotyped with the Ion AmpliSeq method. The geographic origin of the reference strains PRU and CZ-H3 not genotyped with the Ion AmpliSeq method is also included. Thirteen non-European isolates, also genotyped with the Ion AmpliSeq method are excluded. Details are described in Supplementary Table 1

In total, 170 *T. gondii* specimens, including 123 cell-culture isolates and 47 clinical samples, were analyzed according to the workflow depicted in Fig. 1 (also see Supplementary Figure 1). The sample set comprised specimens (Supplementary Table 1) from 19 different countries on the European continent (Fig. 2) and seven non-European countries or locations. Clinical samples originated from 12 different matrices and 15 animal species, including domestic, wild-living and zoo animals. Isolates were cell-cultured as described [17, 19, 20]. The isolates cultivated at Friedrich-Loeffler-Institut (FLI) included the type I reference strains RH_FLI_ and GT1_FLI_, the type II references ME49_FLI_ and NTE_FLI_ and the type III reference NED_FLI_. DNA was extracted by standard methods from cellular pellets or clinical material (Supplementary Table 1). A real-time PCR targeting TgREP-529 [21] was used to characterize DNAs quantitatively [22] (Supplementary Note 1).

### Genotyping by MS analysis and PCR-RFLP

All specimens were genotyped using 15 MS markers [10]. For the markers N60, M102 and AA, the fluorophore (FL) Atto550_Fl_ was used instead of NED_Fl_ for primer labelling. The reported fragment sizes of these three markers were numerically adjusted based on published guidelines [11]. Furthermore, all isolates were genotyped using nine PCR-RFLP markers [12, 23]. In both methods, the reference strains RH_FLI_, ME49_FLI_ and NED_FLI_ were used as positive controls and water as a negative control. The PCR-RFLP and MS genotypes of the reference strains PRU and CZ-H3 described in the literature (Supplementary Table 1) were included in the data analysis.

### Generation of whole genomes and sequence analysis

Whole genome sequencing of 59 *T. gondii* isolates (Supplementary Table 1) was conducted with the Illumina NovaSeq 6000 system in 150 bp paired-end mode (Biodiversa s.r.l., Treviso, Italy). Raw read data of these genomes was processed bioinformatically with 21 publicly available and three reference genomes, ME49, PRU and CZ-H3 (accession numbers in Supplementary Table 1) as described in detail in Supplementary Note 2. Finally, genetic variants were detected and converted into genomic variant call format (gVCF) for further use.

### Sanger sequencing

HPRs (*n* = 55) identified in the gVCF files of the WGS analysis were assessed by Sanger sequencing (details in Supplementary Note 3 and Supplementary Table 2). Sanger HPR sequences obtained in this study for three representative isolates and ME49_FLI_ were aligned to a publicly available ME49 sequence (ToxoDB release 47), using the software Geneious Prime (version 2021.0.1), and SNPs detected by Sanger sequencing were compared to those identified by WGS analysis.

### Library preparation and Ion AmpliSeq sequencing

Library preparation was performed using the Ion AmpliSeq™ Library Kit Plus and IonCode™ Barcode Adaptors (Thermo Fisher Scientific, Waltham, MA, USA), following the manufacturer’s instructions.

For the initial multiplex PCR, an Ion AmpliSeq™ custom panel was designed (Ion AmpliSeq Designer, version 7.49, Thermo Fisher Scientific) by using a ME49 genome sequence (ToxoDB release 47), a BED file containing information about genetic variants of a subset (*n* = 43) of all available *T. gondii* genomes (Supplementary Note 2) and the locations of 24 Sanger sequencing confirmed target regions. Since six regions were excluded by the Ion AmpliSeq Designer, the panel consisted of 68 primers divided into two pools covering 18 regions (Supplementary Table 3). The final target regions were larger compared to the target regions initially identified by WGS analysis or covered these only partially (T16, T32, T51), as illustrated in Supplementary Figure 2a–b. PCR cycling conditions were 99 °C for 2 min, followed by 24, 26 or 28 cycles at 99 °C for 15 s and 60 °C for 8 min.

Adapter ligation was followed by size selection as described [24]. Library quality was checked using the High Sensitivity DNA Kit on an Agilent 2100 Bioanalyzer (Agilent Technologies, Santa Clara, CA, USA) or by using the 4200 TapeStation (Agilent Technologies). Libraries were quantified with the QIAseq™ Library Quant Assay (Qiagen Sciences, Germantown, MD, USA), pooled including the Ion S5 Calibration Standard and the pools sequenced on an Ion 530 chip with an Ion S5 XL System (Thermo Fisher Scientific) in 400 bp-mode according to the manufacturer’s instructions.

### Data analysis of the Ion AmpliSeq sequencing results

For data analyses, a reference sequence set (accessible at https://zenodo.org/; DOI: 10.5281/zenodo.8377016), in the following referred to as AmpliSeq-ME49-Reference, was created using the sequence data of each target region in the genome of ME49 (ToxoDB release 53) with additional 10 bp added to the 5′- and 3′-ends of the regions. The final target region corresponded in 17 of 18 targets to the amplicons generated by the primer panel as described in Supplementary Figure 2a–b and Supplementary Table 4. In the case of T26, the final target region was shortened for data analysis, because runs of consecutive thymine nucleotides (poly[T]) had led to ambiguous sequencing results**.** Sequence reads of each library were analyzed by reference mapping with the Torrent Mapping Alignment Program (TMAP-ion, version 3.4.0) to generate bam files for further use. Mapping quality (MQ) of the reads against the AmpliSeq-ME49-Reference was analyzed by Qualimap bamqc (v2.3) [25]. Furthermore, several BCFtools (v1.15.1 [using htslib 1.16]) [26] were employed, including the “mpileup” command to call variants in each Ion AmpliSeq record with mapped reads to generate library-specific variant call format (VCF) files. All Ion AmpliSeq VCF files were merged into a multiVCF file using the “bcftools merge” command and all variants were filtered by VCFtools (v0.1.16-20) using the hard filter criteria of MQ > 30 and read depth (DP) > 10. Moreover, the combination of Samtools faidx and the “bcftools consensus” command [26] was used to convert the VCF data into the FASTA format. The program Snp-sites (2.5.1) [27] was applied to extract the variable sites from the FASTA sequences. If parts of the AmpliSeq-ME49-Reference were not covered by the reads of specific genotypes, the corresponding nucleotides were indicated as “N” in the FASTA file. The aligned FASTA file containing respective library-specific SNPs was then converted into the NEXUS format and incorporated into SplitsTree4 software (version 4.18.1) [28] to generate unrooted phylogenetic networks using a neighbour-net method and 1000 bootstrap replicates. Sample IDs were replaced by numbers (Supplementary Table 1).

To verify the results of the automated analysis described above, the sequence reads of each library were also mapped to a ME49 genome (ToxoDB release 53) using the Geneious Prime mapper (version 2021.0.1) and default settings. Coverage of the target regions was analyzed, and positions of potential SNPs were visually inspected.

### Assessing the sensitivity of the Ion AmpliSeq method

The analytical sensitivity of the Ion AmpliSeq method was assessed with a set of serially diluted DNA of ME49_FLI_ previously used in a ring trial to harmonize MS typing [11]. The three dilutions were characterized by real-time PCR with Ct values of 23.79, 27.43 and 30.26, corresponding to DNA concentrations of 1 ng/μl, 0.1 ng/μl and 0.01 ng/μl. Each dilution was amplified with 24, 26 and 28 cycles in the Ion AmpliSeq multiplex PCR. The experiment was repeated three times.

### Genotyping of specimens with the Ion AmpliSeq method

Library preparation of 170 specimens was performed as described above. Based on the results of the sensitivity assessment, 24 cycles were defined as the standard protocol, but five isolates with Ct values > 24.5 and all clinical samples with Ct values > 22.0 were amplified with 28 cycles (Supplementary Table 1).

## Results

### MS and PCR-RFLP typing results

**Fig. 3:**
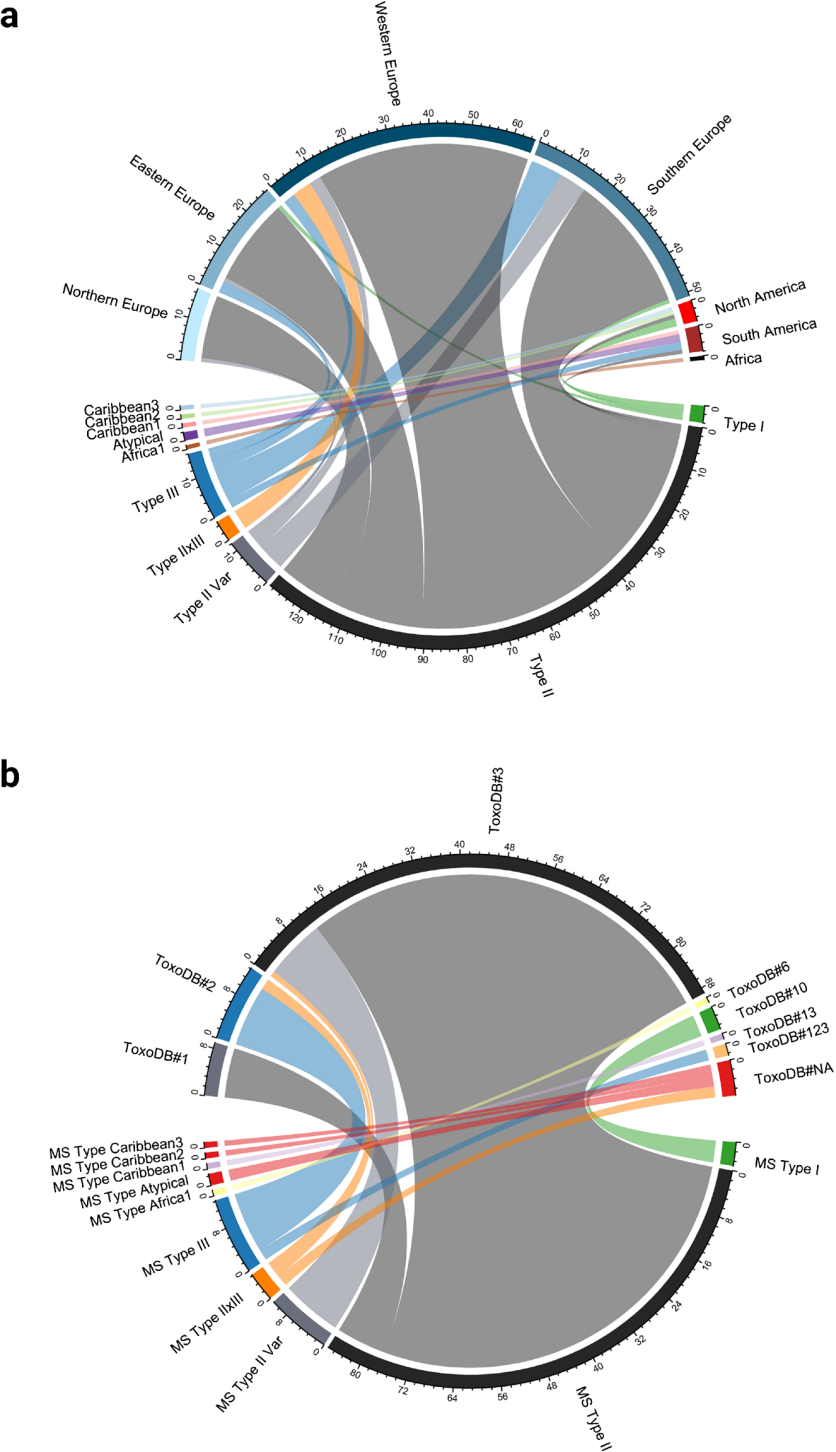
Microsatellite (MS) typing of *T. gondii* specimens using 15 markers. **a** Ten different categories of MS genotypes were reported for DNA from 170 specimens genotyped in this study and additionally for MS genotypes of the reference strains PRU and CZ-H3 described in the literature in relation to their regional origin. Seven regions were defined, consisting of Northern Europe (Denmark, Finland, Sweden, Norway), Eastern Europe (Austria, Czech Republic, Poland, Romania, Serbia, Slovakia), Southern Europe (Greece, Italy, Portugal, Spain), Western Europe (France, Germany, Netherlands, Switzerland, UK), Africa, North America, and South America. **b** Ten different categories of MS genotypes were reported for 123 isolates genotyped in this study and MS genotypes of the reference strains PRU and CZ-H3 described in the literature in relation to their PCR-RFLP genotyping results

By MS typing, most specimens (*n* = 129) were genotyped as type II (Fig. 3, Supplementary Table 1). In addition, twelve specimens were categorized as type II variants as they showed a deviation on one MS lineage typing marker. Eight were W35, two TgM-A variants and one specimen each was a XI.1 or a B18 variant. Four specimens belonged to type I, 16 to type III and five specimens were categorized as type II × III recombinants. Furthermore, the six non-archetypal strains were classified as Africa 1, Caribbean 1, Caribbean 2, Caribbean 3 and Atypical.

Nine of the 86 type II isolates were PCR-RFLP genotyped as ToxoDB#1, while the remaining 77 type II and all type II variant isolates belonged to ToxoDB#3 (Fig. 3, Supplementary Table 1). MS type I corresponded to ToxoDB#10 and 11/13 type III isolates belonged to ToxoDB#2. Two type III isolates from Argentina were classified as ToxoDB#123 by PCR-RFLP. One of the type II × III recombinant isolates belonged to ToxoDB#3 and two to ToxoDB#2. The two remaining recombinants and the isolates MS typed as atypical, Caribbean 2 and 3 could not be assigned to any known PCR-RFLP ToxoDB number. MS type Africa 1 corresponded to ToxoDB#6 and Caribbean 1 to ToxoDB#13.

### Identification of HPRs in the nuclear genome of *T. gondii*

WGS data of 43 *T. gondii* type II genomes (Supplementary Table 1, Supplementary Figure 3) were used for the identification of HPRs (Supplementary Table 5). The mean number of reads per library was 62.1 M (range, 29.9–620.6 M) and the median depth of coverage after mapping to the ME49 genome, in the following referred to as ME49 reference, was 1077× in the case of ME49 (SRR6793863) and 15×–338× for the remaining 42 isolates. An average of 98.1% ± 1.4% standard deviation of each genome was mapped with over 10× coverage.

**Fig. 4:**
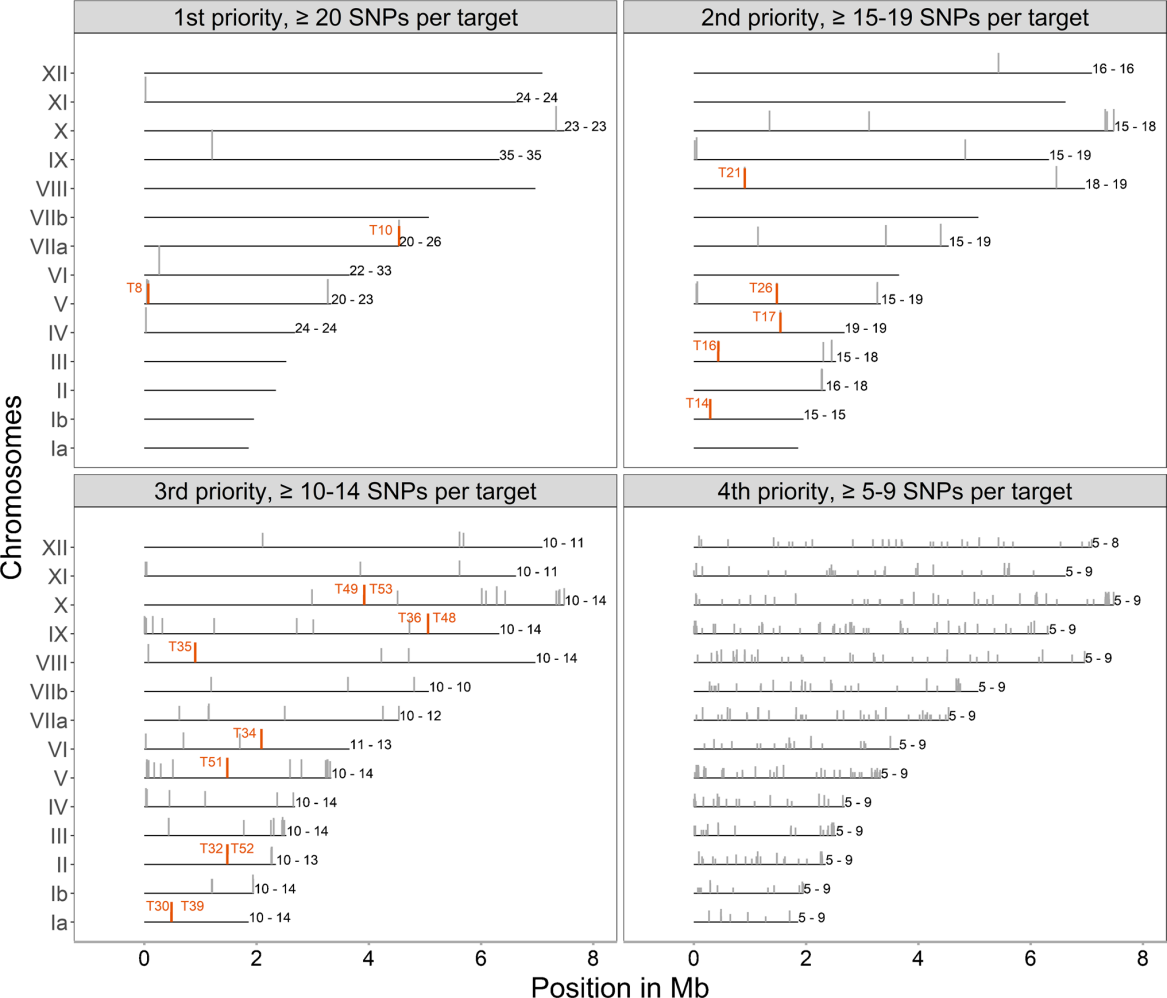
SNP maps of all 14 *T. gondii* chromosomes based on the numbers of SNPs detected in non-overlapping windows of 333 bp in 43 type II genomes relative to the genome of ME49 (ToxoDB release 47). All identified highly polymorphic regions were categorized as first, second, third or fourth priority targets and their positions on the chromosomes are indicated with grey bars. Minimum and maximum numbers of SNPs per target are indicated on the right side of each chromosome. The 18 target regions used for Ion AmpliSeq typing are shown in orange

When mapping to ME49 reference, the SNPs found in the analyzed 43 *T. gondii* genomes sum up to a total of 65,006. The SNPs were used to identify target regions for the Ion AmpliSeq method (Table 1). SNPs were counted in non-overlapping windows of 333 bp, and four prioritization categories of target regions were defined. Nineteen target regions (Fig. 4) were categorized as first priority (20–35 SNPs), 37 as second priority (15–19 SNPs), 136 as third priority (10–14 SNPs) and 673 as fourth priority (5–9 SNPs). The first priority targets were located on seven chromosomes, mainly in subtelomeric regions. The second priority targets were located on ten chromosomes, while third and fourth priority targets were distributed all throughout the chromosomes (Fig. 4, Supplementary Table 5).

**Table 1:** Number of SNPs in 43 *T. gondii* type II whole genomes compared to a ME49 genome (ToxoDB release 47) in non-overlapping windows of 333 bp of the genome. Four prioritization categories of target regions for the new Ion AmpliSeq-based typing method were defined based on the number of SNPs

Chromosome	Number of SNPs relative to ME49 in 43 *T. gondii* type II whole genomes per 333 bp windows					Total	SNPs/10 kb
	1–4	5-9 (4^th^ priority targets)	10-14 (3^rd^ priority targets)	15-19 (2^nd^ priority targets)	20-35 (1^st^ priority targets)		
Ia	1158	11	3	0	0	1172	6.30
Ib	1643	20	4	1	0	1668	8.53
II	1717	38	9	3	0	1767	7.53
III	1962	50	19	5	0	2036	8.04
IV	2145	35	8	1	1	2190	8.15
V	2677	68	29	11	9	2543	6.95
VI	2508	28	5	0	2	3858	8.49
VIIa	3784	60	6	4	4	3821	7.54
VIIb	3783	35	3	0	0	5543	7.95
VIII	5491	41	9	2	0	4803	7.59
IX	4704	77	18	3	1	2794	8.39
X	5622	132	15	6	1	5776	7.72
XI	4729	41	5	0	1	4776	7.21
XII	4804	37	3	1	0	4845	6.83
Total	46,727	673	136	37	19	47,592	8.00

### Confirmation of WGS findings using Sanger sequencing

Sanger sequencing confirmed the WGS data of 24/55 tested SNP dense regions (Supplementary Table 6). Of the sequenced targets, 15.4% (2/13) of first priority, 50.0% (8/16) of second priority, 52.1% (12/23) of third priority and 66.7% (2/3) of the fourth priority targets were confirmed. SNP analysis was not possible for 13/55 regions, due to overlapping peaks in the Sanger sequences resulting in low Phred quality scores. Furthermore, the sequences of 4/55 regions were too short to cover the whole region after mapping to ME49, and in the case of 10/55 regions, no SNPs were detected by Sanger sequencing or observed SNPs were not in accordance with WGS data. The Ion AmpliSeq primer panel was designed using all 24 confirmed regions, containing 336 different SNPs. Six of the confirmed 24 regions were excluded from primer design as detailed in Methods.

### Establishment of the Ion AmpliSeq method

#### Analytical sensitivity of the Ion AmpliSeq method

The analytical sensitivity of the Ion AmpliSeq method was assessed with serially diluted DNA of ME49_FLI_. All libraries were generated with an Ion AmpliSeq primer panel that included the 18 confirmed target regions located on 11 chromosomes (Supplementary Table 4). The mean number of reads per library was 261,220 and an average of 97.8% (range 90.3–99.8%) of the AmpliSeq-ME49-Reference was covered ≥ 30× by each library after mapping (Supplementary Table 7).

**Fig. 5:**
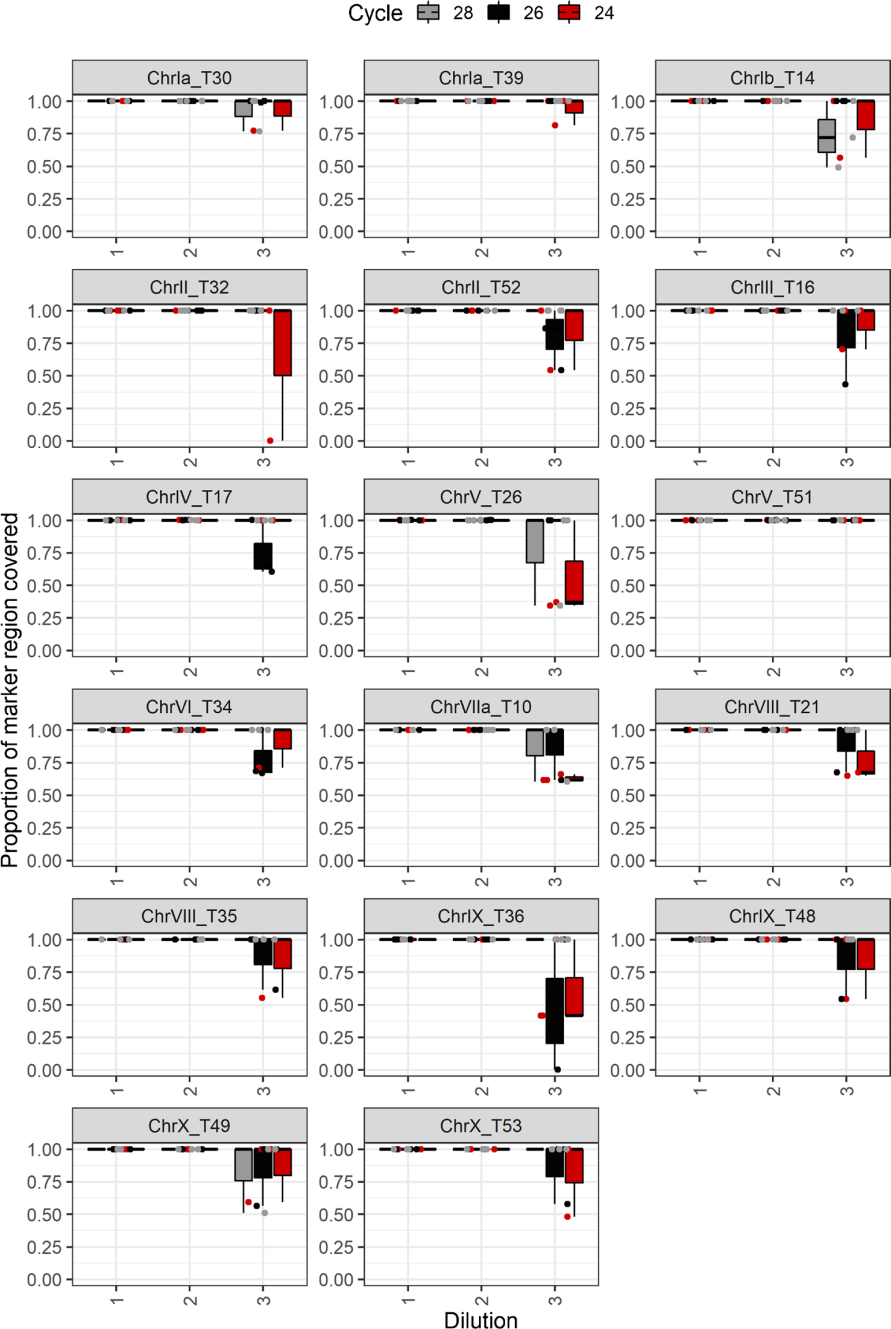
Coverage of 17 target regions by ME49 replicates used to assess the analytical sensitivity of the Ion AmpliSeq method after mapping to mapped the genome of ME49 (ToxoDB release 53). The proportion of coverage of each region is shown in relation to the different dilutions (1, 2, and 3 correspond to *T. gondii *DNA concentrations of 1 ng/μl, 0.1 ng/μl and 0.01 ng/ μl) and the number of PCR cycles

The coverage per target region was analyzed based on alignments with the entire ME49 genome. Target region T8 was identified as repetitive, since the reads were mapped to a length of about 10 kb of the reference genome instead of the expected 600 bp. Therefore, this region was excluded from further analyses. The reads of the first and second dilution covered all 17 remaining regions in each of the replicates, regardless of the number of cycles in the multiplex PCR (Table 2, Fig. 5). Reduced coverage completeness was observed in the third dilution of the samples. In addition, we found that 9–12 regions were completely covered if 24 cycles were used, while using 28 cycles increased the complete coverage up to 14–16 regions.

**Table 2:** Number of regions completely covered by the ME49 replicates used to assess the analytical sensitivity of the Ion AmpliSeq method after mapping to a ME49 reference genome (ToxoDB release 53). Coverage is shown in relation to the different dilutions and number of cycles used in the Ion AmpliSeq multiplex PCR

Dilution	PCR cycles	Completely covered regions, relative to a ME49 reference genome		
		ME49 Ion AmpliSeq replicate 1	ME49 Ion AmpliSeq replicate 2	ME49 Ion AmpliSeq replicate 3
10(-1)	24	17	17	17
10(-1)	26	17	17	17
10(-1)	28	17	17	17
10(-2)	24	17	17	17
10(-2)	26	17	17	17
10(-2)	28	17	17	17
10(-3)	24	12	10	9
10(-3)	26	14	13	9
10(-3)	28	16	15	14

#### General sequencing results of specimens

In total, 170 libraries comprising the set of the 17 final target regions were generated with the Ion AmpliSeq primer panel. Six libraries (TgShSp12-15, TgShSp18 and TgShSp19) were excluded from analysis after mapping to the AmpliSeq-ME49-Reference, since their sequences did not completely cover any of the target regions with a DP > 10. The Ion AmpliSeq results of 164 remaining libraries were analyzed (Supplementary Table 8).

The mean number of reads per library was 183,937 (range, 9279–1,323,246). More than 99.0% of the reads per library could be mapped to the AmpliSeq-ME49-Reference. Overall, the median depth of coverage was 4324×. Furthermore, an average of 97.5% of the AmpliSeq-ME49-Reference was covered ≥ 30× by the reads of type II samples, 94.7% by type II × III and type III, 93.6% by Caribbean 1-3, 81.2% by type I, 79.7% by Africa 1 and 77.9% by the reads of the atypical specimens (Supplementary Figure 4).

The coverage of each target region was analyzed based on alignments with the entire ME49 genome. Most regions were covered by the reads of the libraries regardless of the genotype (Supplementary Figures 5a–b). Sequencing of target region T14 failed partially or completely in case of type I and atypical isolates. Sequencing of target region T30 failed in case of Africa 1. In addition, reads for the target regions T21 and T35 were missing in case of type I, Africa 1, Caribbean 1, Caribbean 2 and atypical specimens.

#### SNPs of *T. gondii* type II isolates detected by the Ion AmpliSeq method compared to WGS data

The results of type II isolates were compared to their WGS data, if available, for the validation of SNPs detected by the Ion AmpliSeq method relative to ME49 (Table 3, Supplementary Table 9). For simplification, only the number of SNPs is described, as the majority of SNPs identified by both methods (WGS, AmpliSeq) were located at the same positions. The minimum number of SNPs per isolate was the same for both methods and ranged between zero and six per region. The maximum number of SNPs per isolate detected by WGS was higher in the case of three regions compared to Ion AmpliSeq.

**Table 3:** Comparison of the number of SNPs detected by Ion AmpliSeq typing and whole genome sequence (WGS) analysis relative to ME49 (ToxoDB release 53). For this comparison, 78 *T. gondii* type II isolates were used. An equal minimum or maximum number of SNPs implies that the SNPs are located at the same positions as well as the number of different SNPs per region considers the SNP positions

Chromosome	Target region	Minimum number of SNPs per isolate		Maximum number of SNPs per isolate		Number of different SNPs per region	
		Ion AmpliSeq	WGS	Ion AmpliSeq	WGS	Ion AmpliSeq	WGS
Ia	T30	0	0	5	5	20	20
Ia	T39	0	0	7	7	18	18
Ib	T14	0	0	7	7	28	28
II	T32	0	0	5	5	12	14
II	T52	1	1	6	6	17	18
III	T16	0	0	8	9	19	23
IV	T17	6	6	12	12	19	19
V	T26	0	0	5	6	13	17
V	T51	0	0	6	6	13	16
VI	T34	0	0	8	8	19	17
VIIa	T10	0	0	9	25	11	27
VIII	T21	0	0	4	4	23	23
VIII	T35	0	0	5	5	26	25
IX	T36	0	0	3	3	16	15
IX	T48	0	0	7	7	22	21
X	T49	0	0	7	7	16	16
X	T53	0	0	7	7	18	20

Comparing the total number of different SNPs per region, considering also the SNP positions, revealed further differences between both analyses. In six regions (T30, T39, T14, T17, T21, T49), no differences were detected between Ion AmpliSeq and WGS data. In seven regions, WGS analysis revealed a larger number of SNPs, mainly because some SNPs detected in the Ion AmpliSeq data were excluded due to the filter criterion MQ > 30. One or two additional SNPs were observed with the Ion AmpliSeq method in the remaining four regions (details in Supplementary Table 9).

#### Separation of different *T. gondii* genotypes

**Fig. 6:**
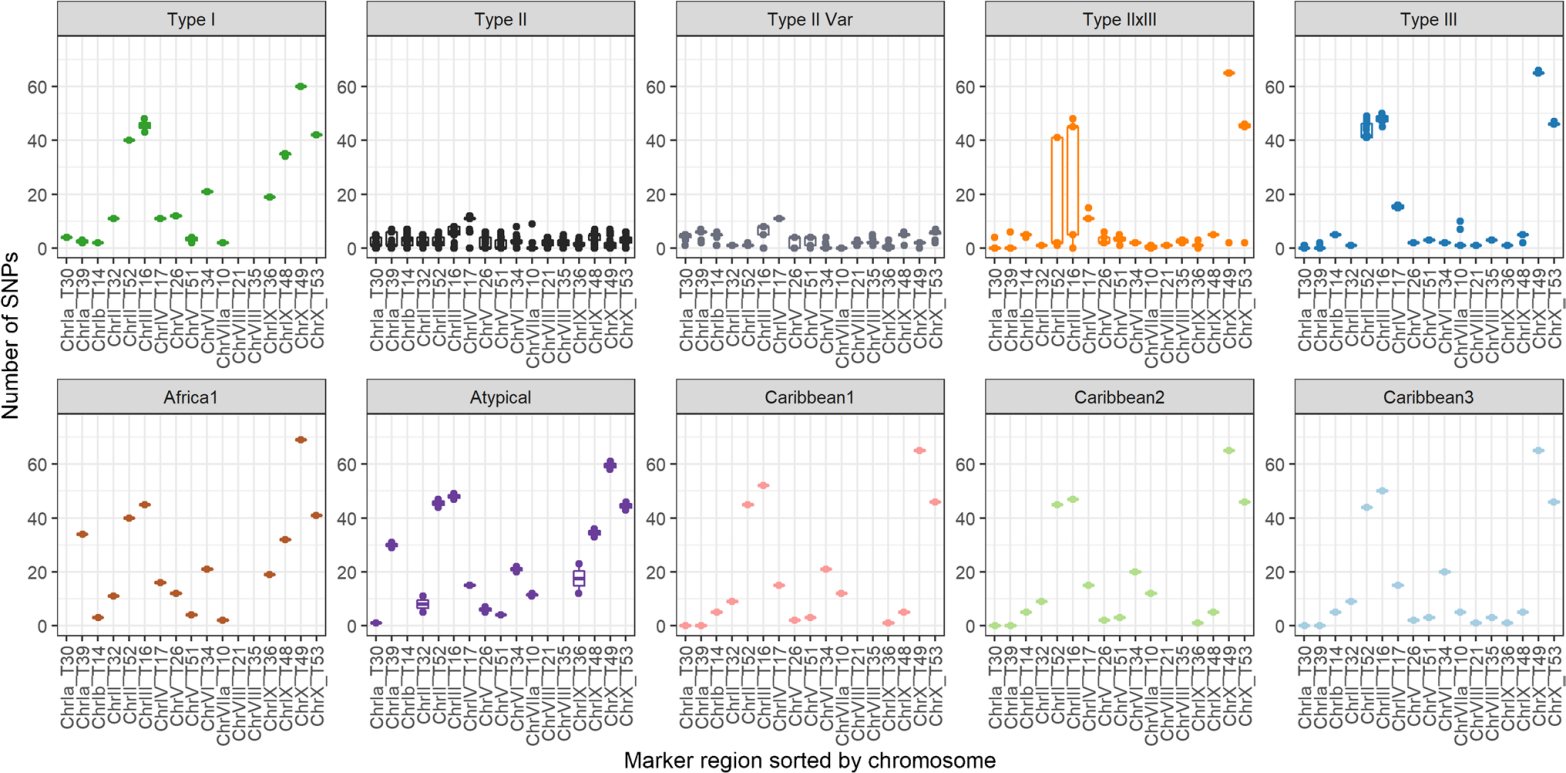
Comparison of the numbers of SNPs detected by Ion AmpliSeq typing in *T. gondii* specimens relative to the AmpliSeq-ME49-Reference per region and per genotype. The numbers of specimens per genotype are not equally distributed. The figure includes results of 121 type II specimens, twelve type II variants, 14 type III (excluding C25 and C26, classified as ToxoDB #123 by PCR-RFLP typing) and four type I specimens. Only one specimen each was analyzed in case of Africa 1 and Caribbean 1, 2 and 3 and in addition, two atypical specimens and five type II × III recombinants were examined. If a boxplot is missing, the affected region was not covered by the reads of the respective genotype and no SNPs could be reported. This was the case for Africa 1 in target region T30 as well as for the atypical specimens in target region T14. In addition, target regions T21 and T35 were not covered by the reads of type I, Africa 1, Caribbean 1, Caribbean 2 and atypical specimens

For testing the discriminatory power of the Ion AmpliSeq method among different genotypes, the number of SNPs detected per region relative to the AmpliSeq-ME49-Reference in 164 libraries (Supplementary Table 8) and their positions (Supplementary Table 10) were compared. In 7/17 target regions, the results were similar (Fig. 6, Supplementary Figure 6). Moreover, the SNPs of type III and Caribbean 1, 2 and 3 were consistent across the target regions T36 and T48 and type III and Caribbean 3 were identical across the target regions T21 and T35. The non-type II genotypes revealed a noticeably larger number of SNPs than type II in the target regions T52, T16, T49 and T53. In addition, type I and Africa 1 showed a large number of SNPs in the target regions T36 and T48.

**Fig. 7:**
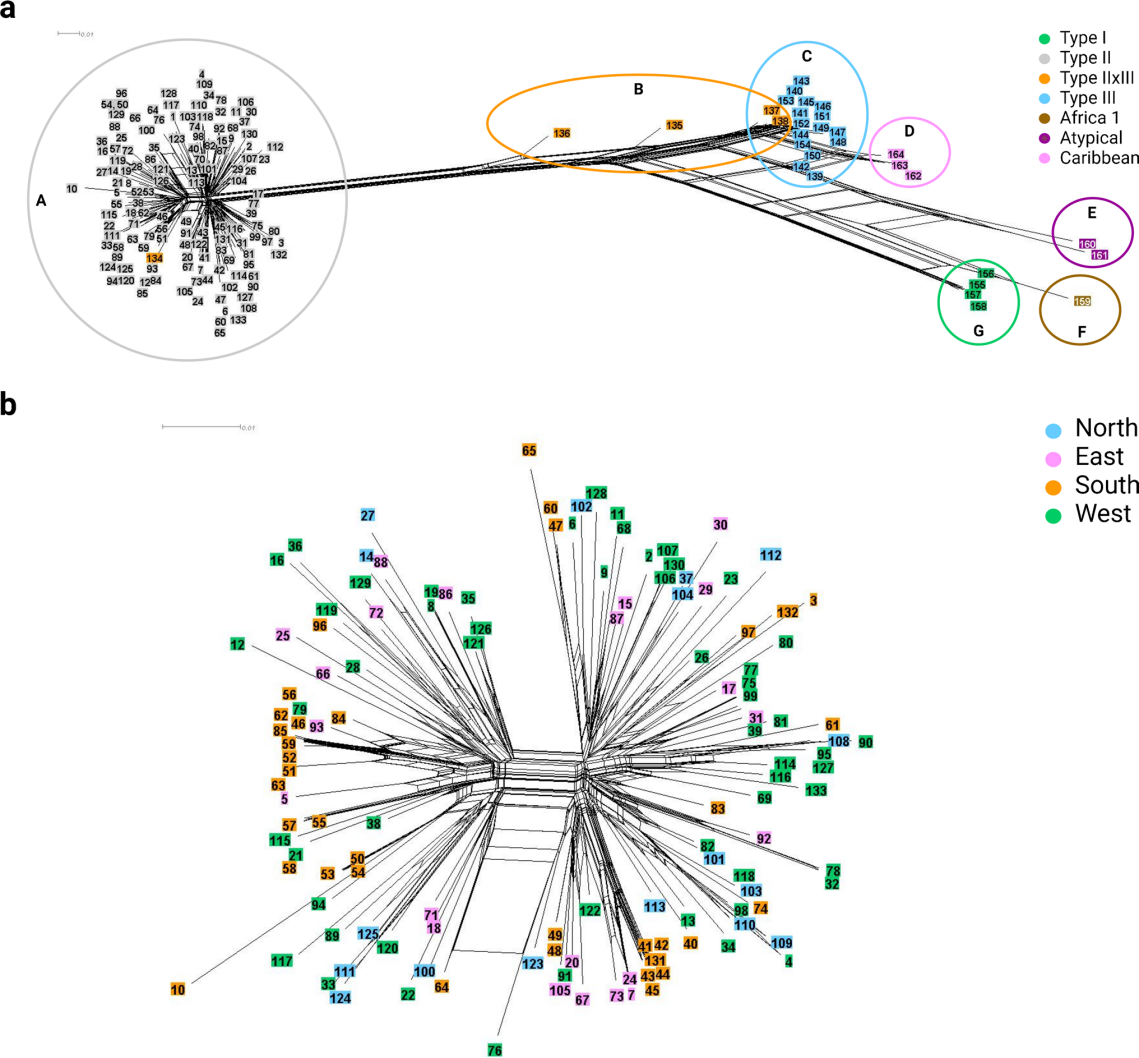
Neighbour-net analysis of *T. gondii* specimens based on SNPs detected by Ion AmpliSeq typing relative to the AmpliSeq-ME49-Reference in 17 target regions (software SplitsTree4). **a** Analysis of 164 specimens belonging to different genotypes revealed seven groups. All type II specimens are located in group A, type III in group C and type I in group G. Four type II × III recombinant strains (coloured in orange) are in group B and one in group A. Group D is represented by the genotypes Caribbean 1, 2 and 3, group E by two atypical strains and one specimen typed as Africa 1 is located in group F. **b** Analysis of 131 European type II specimens distinguishing specimens from North, East, South and West Europe. No clear regional patterns can be observed. However, different passages from the same isolates were identified as identical (No. 7 and 24; No. 8 and 19; No. 32 and 78) as were specimens from abortion outbreaks (No. 42–45 and 131; No. 48 and 49). Furthermore, eight specimens (No. 46, 51, 52, 56, 59, 62, 79, 85), which showed the same variation in the MS marker W35, were identical or very similar

In summary, based on these differences in SNP density and positions, several genotypes could be clearly distinguished by the Ion AmpliSeq method, which was also confirmed by neighbour-net analysis (Fig. 7). Caribbean 1, 2 and 3 resembled type III, while Africa 1 was more similar to type I. Furthermore, one of the five type II × III recombinant specimens could not be differentiated from type II and two other recombinants were similar to type III. The results of the remaining two recombinant specimens identified them as a mix of type II and type III.

#### Ion AmpliSeq fingerprinting of *T. gondii* type II specimens compared to MS typing

All 131 sequenced and analyzed European type II specimens, including MS type II variants, were used to test the ability of the Ion AmpliSeq method to detect intra-genotype variability. The analysis was based on their SNPs relative to the AmpliSeq-ME49-Reference and the results were visualized by neighbour-net analysis (Fig. 7). Both, Ion AmpliSeq and MS typing, differentiated the same number of profiles (*n* = 115), of which 107 were unique. Eight profiles were detected in two or more libraries. Of three isolates, DNAs of two different passages were analyzed, which revealed identical results by both methods (SplitsTree No. 7 and 24, 8 and 19, 32 and 78). Furthermore, No. 42–45 and 131 could not be differentiated, which was also true for No. 48 and 49. In both cases, the specimens originated from an abortion outbreak in a sheep flock. Moreover, No. 50, 53 and 54 were identical in both typing methods; they were all from adult sheep of the same farm. The specimens No. 55, 57 and 58 were also identical in the Ion AmpliSeq results; No. 57 and 58 originated from the same farm. Interestingly, No. 55 was different in MS typing as compared to specimens No. 57 and 58. In addition, out of eight specimens, classified as MS type II variants as they showed a deviation in the MS marker W35, five specimens (No. 46, 51, 56, 59 and 79; the first four originating from Spain and the remaining from France) were not differentiated by the Ion AmpliSeq method. In contrast, only the first three specimens (No. 46, 51, 56) had exactly the same profile by MS typing. On the other hand, three of the eight type II variants (No. 52, 62, 85), which had the same MS profile, were differentiated by the Ion AmpliSeq method.

## Discussion

Genotyping of *T. gondii* is important to differentiate circulating strains, trace infection sources in outbreaks and characterize strains causing particular clinical forms of disease [17, 29]. Given the association of genotypes found in Central and South America with higher virulence and greater clinical relevance [18, 30], genotyping is also important to detect the introduction of genotypes into new areas such as Europe.

In this study, we aimed to develop an NGS-based typing method with high typing resolution among closely related type II strains, which may allow for automated and standardized data analysis.

Most European specimens were classified as type II by MS typing, which reflects the parasite (clonal) population structure in Europe [8]. The non-archetypal specimens originated from Central and South America and from Africa, where the population structure is much more diverse [7, 31].

WGS data of 43 *T. gondii* type II isolates provided the base for the identification of HPRs when mapped to the genome of ME49. Approximately 6–8.5 SNPs per 10 kb were detected per chromosome. This corresponds to previous findings for type II, where about 10 SNPs per 10 kb were observed on all chromosomes [9, 17].

Fifty-five out of the 865 identified SNP dense regions were explored further using Sanger sequencing, as this study focused on the identification of genomic regions that were located on different *T. gondii* chromosomes that simultaneously harboured a large number of SNPs. This demonstrates the future potential to add additional targets to the Ion AmpliSeq primer panel. Some of the 55 tested regions were not confirmed by Sanger sequencing. Their majority, especially the first priority targets, was located in subtelomeric chromosomal regions. Subtelomeres are often affected by recombination events and thus repetitive sequence rich [32], which can cause sequencing problems.

In our study, the most frequent causes of inconclusive Sanger sequencing were overlapping peaks resulting in insufficient sequence quality. This may be due to the presence of repetitive sequences or multiple priming sites in the DNA template [33]. Discrepancies between SNPs detected by Sanger sequencing compared to WGS and the absence of expected SNPs in the Sanger sequences may also be explained by repetitive regions, as incorrect mapping of reads to the reference may lead to the identification of spurious SNPs.

The analytical sensitivity of the Ion AmpliSeq method was assessed with serial dilutions of ME49 DNA prepared for a previous ring trial [11]. Using the same set of dilutions ensured comparable results. Overall, the analytical sensitivity is comparable to MS typing, as failure of individual markers was only observed in the 3rd dilution with both methods [11]. Even in the 3rd dilution, at least half of the regions were completely covered by the Ion AmpliSeq sequences and failing regions were partially covered in most cases. Furthermore, increasing the number of cycles in the multiplex PCR improved coverage. This adjustment may help in the analysis of low concentrated samples. Nevertheless, it has to be noted that the use of too many cycles may increase nonspecific amplification and amplification errors. Therefore, we defined 24 cycles as the standard protocol, but used 28 cycles for specimens with higher Ct values.

For the validation of the Ion AmpliSeq results, SNPs detected in type II isolates were compared to those determined with WGS analysis. A few differences were observed when comparing the total number of different SNPs per region, mainly because SNPs detected in the Ion AmpliSeq data were excluded due to a MQ ≤ 30. It has to be considered that previous studies described lower quality scores for Ion Torrent bases compared to Illumina bases [34–36]. Since the base quality score affects the calculation of the MQ, this can also cause a higher MQ in the Illumina than in the Ion Torrent data. However, using a lower MQ as a filter criterion to adapt the Ion AmpliSeq results to WGS data bears the risk of detecting false positive SNPs.

Seven genotypes, two atypical and two recombinant specimens could be clearly distinguished by Ion AmpliSeq typing, due to differences in SNP density and positions relative to ME49. Sequencing of a few target regions failed partially or completely in case of type I, Africa 1, Caribbean 1, Caribbean 2 and the atypical specimens. In a neighbour-net analysis, type III showed less genetic distance to type II than type I to type II. Furthermore, Caribbean 1, 2 and 3 were grouped close to type III, while Africa 1 was more similar to type I as found in earlier studies on genetic distances [9, 17].

**Fig. 8:**
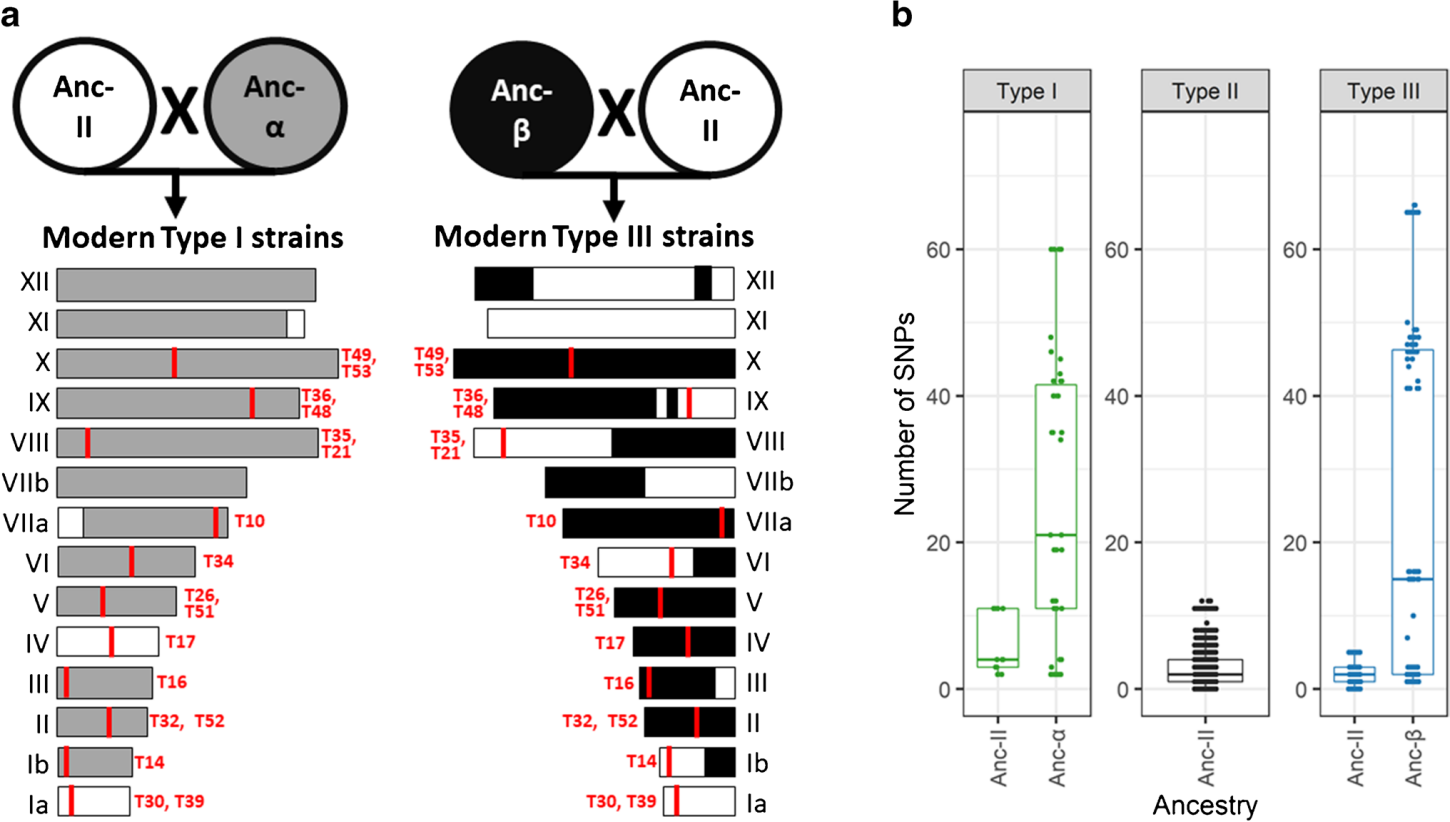
Proposed genealogy of the *T. gondii* lineages type I and III and chromosome segregation during the proposed crosses, modified from Boyle et al. (2006) [37], combined with the number of SNPs detected by Ion AmpliSeq typing. **a** Chromosome segregation during the two proposed crosses (ancestral type II (Anc-II) × ancestral α (Anc-α) and ancestral type II × ancestral β (Anc-β)) (details in Supplementary Figure 4a-n). On the left (for type I) and right (for type III), all 14 chromosomes are represented schematically with their proposed ancestry coloured in grey (α), black (β), or white (type II). The positions and the names of the 17 Ion AmpliSeq target regions on the chromosomes are denoted in red. **b** Number of SNPs detected by Ion AmpliSeq typing within types I, II and III specimens relative to the AmpliSeq-ME49-Reference (details in Supplementary Figure 5). In case of type I and type III, the regions and the associated SNPs were differentiated into ancestral type II (Anc-II) and ancestral α (Anc-α) and ancestral β (Anc-β). Large numbers of SNPs per region are only observed in Ion AmpliSeq targets located in parts of the genome, for which Ancestral α or β origin was proposed

The genealogy of the clonal lineages type I, II and III indicates that types I and III originated from a cross between an ancestral type II strain and one of two ancestral strains, called α or β [37]. Regions, where the number of SNPs detected by the Ion AmpliSeq method was similar between the three clonal lineages, are likely to be of ancestral type II origin (Fig. 8, Supplementary Figure 7a–n, Supplementary Figure 8). If SNPs observed in type I or type III specimens clearly differed from type II, it is assumed that the corresponding regions originated at least in most cases from the ancestral strains, α or β. These previous findings could also explain why the sequencing of three regions failed in type I, as the Ion AmpliSeq primer panel was designed using a type II genome and local variations in the genomes of different genotypes might interfere with primer binding.

The specimens MS typed as Caribbean 1, 2 and 3 were previously characterized as a result of recombination between different ancestral strains [17]. Chromosome VIII of Caribbean 1 and 2 contains more type I origin segments compared to Caribbean 3, where chromosome VIII is dominated by sequences of type III origin. This fits to the fact that sequencing of the Ion AmpliSeq target regions on chromosome VIII failed in Caribbean 1 and 2, while the results of Caribbean 3 were identical with type III. Only two of the five type II × III recombinants were clearly classified as such by the Ion AmpliSeq method. This may be explained by different targets used for MS typing and depend on the site of recombination in the genome.

In total, 115 different profiles were identified by the Ion AmpliSeq method among 131 European type II specimens. MS typing, which served as a reference, distinguished the same number, but not exactly the same specimens. In our study, all specimens classified as identical by both methods were expected to be identical, as they were passages from the same isolates or originated from an abortion outbreak or the same sheep farm. This indicates the ability of the Ion AmpliSeq method to trace back infection sources in outbreaks. Ion AmpliSeq and MS typing revealed discrepancies in the identification of profiles for eight MS type II variants. We argue that these eight type II variants are genetically very similar and that discrepancies are due to the use of different target regions in the two typing methods. The WGS data when available, for these isolates, supported this hypothesis.

Furthermore, the performed neighbour-net analysis of the European type II specimens did not show a clear proximity of specimens originating from the same European region. However, a detailed study of the correlation between genetic differences and the geographic origin of specimens requires specific cluster analyses, which was out of the scope of this study. To validate the results of a cluster analysis based on the data of the new Ion AmpliSeq method, a cluster analysis based on the whole genome sequences of the corresponding isolates is needed.

In conclusion, we established an Ion AmpliSeq method that can distinguish archetypal and non-archetypal genotypes of *T. gondii* and detect intra-genotype variability among European *T. gondii* type II specimens. In addition to DNA extracted from cell-cultured *T. gondii* isolates, parasites present in clinical samples of different matrices and from different animal species were successfully typed, indicating the suitability of the new method for analyzing a large variety of samples. This is a major benefit for investigations using a One Health approach. The Ion AmpliSeq method appears promising for tracing back infection sources in outbreaks and for the detection of recombinant or non-archetypal strains. Automated data analysis makes data interpretation objective. Furthermore, as only a selection of 55 out of 865 identified SNP dense regions within the *T. gondii* genome were further explored in this work, there is a huge potential to add further target regions to the method.

### Supplementary information

ESM 1 Supplementary Note 1-3 providing additional information about material and methods (PDF 193 kb)

ESM 2 Supplementary Figures 1-8 providing data on the establishment of a new high-resolution next-generation sequencing-based method (PDF 3496 kb)

ESM 3 Supplementary Tables 1-10 providing data on the establishment of a new high-resolution next-generation sequencing-based method (XLSX 274 kb)

## Data Availability

The Ion AmpliSeq sequences generated and analyzed as FASTQ files in this study and four additional files (i.e. “AmpliSeq-ME49-Reference.fasta”, “Tgondii_43Genomes_SNPs_INDELs.bed”, “Tgondii_AmpliSeq_Results_SNPs_VCF.gz” and “Tgondii_IonAmpliSeq_Results_SNPs.fasta”) are available at https://zenodo.org/ with the DOI: 10.5281/zenodo.8377016. The data set of Ion AmpliSeq sequences comprises 164 FASTQ files named according to the DNA identifiers of the analyzing laboratory in Supplementary Table 1 as D170242-D170246, D180563, D180565, D180567, D180569, D200108-D200114, D200116-D200122, D200124-D200129, D200209-D200212, D200284-D200289, D200389-D200394, D201429-D201439, D201444, D201445, D201448-D201464, D210002, D212711, D212719, D212822, D212823, D212937, D212938-D212940, D212961, D213025, D213050, D213302, D213370, D213419, D213421, D213426, D213428, D213432, D213580, D213618, D213622, D220012, D220063, D220071, D220083, D220102, D220116, D220176, D220214-D220219, D220221, D220224-D220228, D220234, D220249, D220252, D220254, D220269, D220324, D220526, D220529, D220530, D220537, D220557, D220606, D220758, D220798, D220820, D220856, D220881, D221297, D221302, D221313, D221316, D221362, D221364-D221380, D221394, D221398-D221402, GT1, RH, ME49, NTE, NED. The 59 genomes sequenced in this study are available upon reasonable request at https://zenodo.org/ with the DOIs: 10.5281/zenodo.8214035 and 10.5281/zenodo.8378855 under the accession numbers given in Supplementary Table 1. The Ion AmpliSeq primer panel used for this study can be shared through the corresponding author to all those which have an account at the Ion AmpliSeq Designer home page (https://www.ampliseq.com).
