# Supplementary material for: Genotyping of European Toxoplasma gondii strains by a new high-resolution next-generation sequencing-based method
Source: Eur J Clin Microbiol Infect Dis. 2023 Dec 15;43(2):355–71. doi: 10.1007/s10096-023-04721-7 (PMC10822014; doi:10.1007/s10096-023-04721-7)
Supplement: Supplementary file 1 — Supplementary Note 1-3 providing additional information about material and methods (PDF 193 kb) [file 10096_2023_4721_MOESM1_ESM.pdf]

Supplementary Notes

Title: Genotyping of European *Toxoplasma gondii* strains by a new high-resolution next-generation sequencing-based method

Journal: European Journal of Clinical Microbiology & Infectious Diseases

Authors: M. Joeres, P. Maksimov, D. Höper, S. Calvelage, R. Calero-Bernal, M. Fernández-Escobar, B. Koudela, R. Blaga, M. Globokar Vrhovec, K. Stollberg, N. Bier, S. Sotiraki, J. Sroka, W. Piotrowska, P. Kodym, W. Basso, F. J. Conraths, A. Mercier, L. Galal, M. L. Dardé, A. Balea, F. Spano, C. Schulze, M. Peters, N. Scuda, A. Lundén, R. K. Davidson, R. Terland, H. Waap, E. de Bruin, P. Vatta, S. Caccio, L. M. Ortega-Mora, P. Jokelainen, G. Schares

Correspondence address:

Gereon Schares
Friedrich-Loeffler-Institut, Federal Research Institute for Animal Health, Institute of Epidemiology, Greifswald – Insel Riems
E-mail: gereon.schares@fli.de

Supplementary Note 1: DNA quantification by real-time PCR 2

Supplementary Note 2: Whole genome sequence analysis 3

Supplementary Note 3: Primer design and Sanger sequencing 4

References 5

Supplementary Note 1: DNA quantification by real-time PCR

A previously reported real-time PCR targeting the TgREP-529 [1] was used to quantify the extracted *T. gondii* DNA. A final reaction volume of 25 µl was applied, using a commercial master mix (5x PerfeCTa Tough Mix, VWR, Darmstadt, Germany) and 1 µl of template DNA. Amplification was done on a CFX96 instrument (Bio-Rad Laboratories GmbH, Munich, Germany) and real-time PCR results were analyzed using the CFX Maestro software Version 4.1 (Bio-Rad Laboratories). To monitor inhibition in real-time PCR, a heterologous plasmid DNA resembling the enhanced green fluorescent protein (EGFP) gene [2] was used as described before [3].

Supplementary Note 2: Whole genome sequence analysis

The quality of the Illumina NGS data was assessed using the 'fastQC' software package (https://www.bioinformatics.babraham.ac.uk/projects/fastqc/). The respective reads were mapped to a *T. gondii* ME49 reference genome from ToxoDB release 47 (http://ToxoDB.org) with the BWA-MEM mapper [4]. The proportion of the reference genome coverage by the mapped reads of the genomes was calculated using the software package BEDTools [5].

In general, GATK tools [6–8] were used for variant analysis where HaplotypeCaller was used to call variants in each genome. Isolate-specific genomic variant call format (gVCF) files were generated. To prepare the data for joint genotyping using the "GenotypeGVCFs" command, the gVCF files of all isolates were combined using the CombineGVCFs tool. In the next step, hard-filtering for single nucleotide polymorphisms (SNPs) and insertions/deletions (INDELs) was applied as recommended by Van der Auwera et al. (2013) [8]. Filtered variants were annotated with the help of the variant annotation software SnpEff [9] applying the gff (gene-finding format) file of the ME49 *T. gondii* reference strain as described in the software manuals. The SnpSift tool [10], VCFtools [11] and BCFtools [12] were used for data selection, validation, merging, comparing, simple statistics and other manipulations or analyses of the annotated multiVCF file**.** For details we refer to the respective manuals of the tools.

The described mapping and variant analysis was done with n=43 genomes (i.e. 40 *T. gondii* genomes sequenced in this study (Supplementary Table 1) and the genomes of the type II *T. gondii* reference strains PRU (SRR350739), ME49 (SRR6793863) and CZ-H3 (SRR7056296), downloaded from the European Nucleotide Archive (https://www.ebi.ac.uk/ena/browser/home)). The resulting multiVCF file was used for the comparative characterization of SNPs and INDELs within these 43 genomes relative to the reference genome of ME49 (ToxoDB release 47) and for the identification of highly polymorphic regions. Furthermore, the multiVCF file was converted into BED format using the software package BEDTools and the "vcf2bed" command. The resulting BED file (assessible at https://zenodo.org/; DOI: 10.5281/zenodo.8377016; named as "Tgondii_43Genomes_SNPs_INDELs.bed"), contained genomic regions and associated annotations, consisting of the names of the chromosomes, the starting and the ending position of genetic variants. Converting to BED format was necessary to include the information about genetic variants in the design of the Ion AmpliSeq primer panel.

A second analysis was performed including all 59 *T. gondii* genomes sequenced in this study and Illumina raw read whole genome data of 13 recently published *T. gondii* genomes [13] and of eight *T. gondii* genomes downloaded from NCBI GenBank (https://www.ncbi.nlm.nih.gov/genbank/) (Supplementary Table 1). The reference genomes of PRU (SRR350739), ME49 (SRR6793863) and CZ-H3 (SRR7056296) were included in the analysis. The resulting multiVCF file was used for the comparative characterization of SNPs and INDELs within these 83 genomes relative to the reference genome of ME49. This analysis provided the base for comparing the Ion AmpliSeq results generated in this study with whole genome data.

Supplementary Note 3: Primer design and Sanger sequencing

In total, 55 regions were chosen to be tested by Sanger sequencing. Criteria for selection of regions for confirmation by Sanger sequencing were (in the order of importance):

- The number of SNPs per region (regions showing the highest number of SNPs were tested first)
- Location on different chromosomes of the *T. gondii* genome
- Differing SNPs among the isolates

To amplify the selected regions, primers were designed using the software Geneious Prime (version 2021.0.1). In general, two primer pairs per region were purchased from Eurofins Genomics (Ebersberg, Germany). Only one primer pair was ordered for each of the target regions T9, T13 and T27, due to off-target binding of the other designed primers. The expected size of amplification products ranged between 400 bp and 716 bp. Details about the primer pairs are summarized in Supplementary Table 2.

Three different isolates and $ME49_{FLI}$ (Supplementary Table 2) were amplified per region with an endpoint PCR in preparation for Sanger sequencing. Primers were used at a final concentration of 0.4 pmol per µl and dNTPs (Stratec Molecular GmbH, Berlin, Germany) at a final concentration of 250 mM each. Taq polymerase (Platinum™ Taq DNA Polymerase; Invitrogen, Carlsbad, CA, USA) had a final concentration of 1 U/25 µl using the buffer system supplied with the enzyme. The PCR cycling conditions were 94 °C for 5 min; then 94 °C/1 min, 62 °C/1 min, 72 °C/1 min, for 10 cycles; followed by 94 °C/1 min, 58 °C/1 min, 72 °C/1 min for 40 cycles. The PCR ended with a final extension at 72 °C for 10 min.

For Sanger sequencing of the amplification products, bands of the expected size were excised from agarose gels and purified with a commercial kit (NucleoSpin®Gel and PCR Clean-up; Macherey–Nagel, Düren, Germany), following the manufacturer’s instructions. Sequencing was performed using the BigDye Terminator v1.1 Cycle Sequencing Kit (Applied Biosystems, Warrington, UK). The labelled products were purified with NucleoSEQ Columns (Macherey-Nagel) and subsequently sequenced on an ABI 3500 Genetic Analyzer (Applied Biosystems).

The software Geneious Prime was used for analyzing the Sanger sequences. For each region, forward and reverse sequences of the individual isolates were aligned to a ME49 (ToxoDB release 47) target sequence of the particular region. SNPs detected in the Sanger sequences were compared to the corresponding WGS data.

References

1. Talabani H, Asseraf M, Yera H et al. (2009) Contributions of immunoblotting, real-time PCR, and the Goldmann-Witmer coefficient to diagnosis of atypical toxoplasmic retinochoroiditis. J Clin Microbiol 47:2131–2135. https://doi.org/10.1128/JCM.00128-09

2. Hoffmann B, Depner K, Schirrmeier H et al. (2006) A universal heterologous internal control system for duplex real-time RT-PCR assays used in a detection system for pestiviruses. J Virol Methods 136:200–209. https://doi.org/10.1016/j.jviromet.2006.05.020

3. Schares G, Joeres M, Rachel F et al. (2021) Molecular analysis suggests that Namibian cheetahs (*Acinonyx jubatus*) are definitive hosts of a so far undescribed *Besnoitia* species. Parasit Vectors 14:201. https://doi.org/10.1186/s13071-021-04697-3

4. Li H (2013) Aligning sequence reads, clone sequences and assembly contigs with BWA-MEM

5. Quinlan AR, Hall IM (2010) BEDTools: a flexible suite of utilities for comparing genomic features. Bioinformatics 26:841–842. https://doi.org/10.1093/bioinformatics/btq033

6. DePristo MA, Banks E, Poplin R et al. (2011) A framework for variation discovery and genotyping using next-generation DNA sequencing data. Nat Genet 43:491–498. https://doi.org/10.1038/ng.806

7. McKenna A, Hanna M, Banks E et al. (2010) The Genome Analysis Toolkit: A MapReduce framework for analyzing next-generation DNA sequencing data. Genome Res 20:1297–1303. https://doi.org/10.1101/gr.107524.110

8. van der Auwera GA, Carneiro MO, Hartl C et al. (2013) From FastQ data to high confidence variant calls: the Genome Analysis Toolkit best practices pipeline. Curr Protoc Bioinformatics 43:11.10.1-11.10.33. https://doi.org/10.1002/0471250953.bi1110s43

9. Cingolani P, Platts A, Le Wang L et al. (2012) A program for annotating and predicting the effects of single nucleotide polymorphisms, SnpEff: SNPs in the genome of Drosophila melanogaster strain w1118; iso-2; iso-3. Fly (Austin) 6:80–92. https://doi.org/10.4161/fly.19695

10. Cingolani P, Patel VM, Coon M et al. (2012) Using *Drosophila melanogaster* as a model for genotoxic chemical mutational studies with a new program, SnpSift. Front Genet 3:35. https://doi.org/10.3389/fgene.2012.00035

11. Danecek P, Auton A, Abecasis G et al. (2011) The variant call format and VCFtools. Bioinformatics 27:2156–2158. https://doi.org/10.1093/bioinformatics/btr330

12. Li H, Handsaker B, Wysoker A et al. (2009) The Sequence Alignment/Map format and SAMtools. Bioinformatics 25:2078–2079. https://doi.org/10.1093/bioinformatics/btp352

13. Galal L, Ariey F, Gouilh MA et al. (2022) A unique *Toxoplasma gondii* haplotype accompanied the global expansion of cats. Nat Commun 13:5778. https://doi.org/10.1038/s41467-022-33556-7

Funding: This work was part of TOXOSOURCES project, supported by funding from the European Union’s Horizon 2020 Research and Innovation programme under grant agreement No 773830: One Health European Joint Programme.
