# Supplementary material for: Genotyping of European Toxoplasma gondii strains by a new high-resolution next-generation sequencing-based method
Source: Eur J Clin Microbiol Infect Dis. 2023 Dec 15;43(2):355–71. doi: 10.1007/s10096-023-04721-7 (PMC10822014; doi:10.1007/s10096-023-04721-7)
Supplement: Supplementary file 2 — Supplementary Figures 1-8 providing data on the establishment of a new high-resolution next-generation sequencing-based method (PDF 3496 kb) [file 10096_2023_4721_MOESM2_ESM.pdf]

Supplementary Figures

Title: Genotyping of European *Toxoplasma gondii* strains by a new high-resolution next-generation sequencing-based method

Journal: European Journal of Clinical Microbiology & Infectious Diseases

Authors: M. Joeres, P. Maksimov, D. Höper, S. Calvelage, R. Calero-Bernal, M. Fernández-Escobar, B. Koudela, R. Blaga, M. Globokar Vrhovec, K. Stollberg, N. Bier, S. Sotiraki, J. Sroka, W. Piotrowska, P. Kodym, W. Basso, F. J. Conraths, A. Mercier, L. Galal, M. L. Dardé, A. Balea, F. Spano, C. Schulze, M. Peters, N. Scuda, A. Lundén, R. K. Davidson, R. Terland, H. Waap, E. de Bruin, P. Vatta, S. Caccio, L. M. Ortega-Mora, P. Jokelainen, G. Schares

Correspondence address:

Gereon Schares
Friedrich-Loeffler-Institut, Federal Research Institute for Animal Health, Institute of Epidemiology, Greifswald – Insel Riems
E-mail: gereon.schares@fli.de

Supplementary Fig. 1 2

Supplementary Fig. 2a-b 4

Supplementary Fig. 3 5

Supplementary Fig. 4....................6

Supplementary Fig. 5a-b 8

Supplementary Fig. 6 9

Supplementary Fig. 7a-7n 24

Supplementary Fig. 8 25

Funding 26

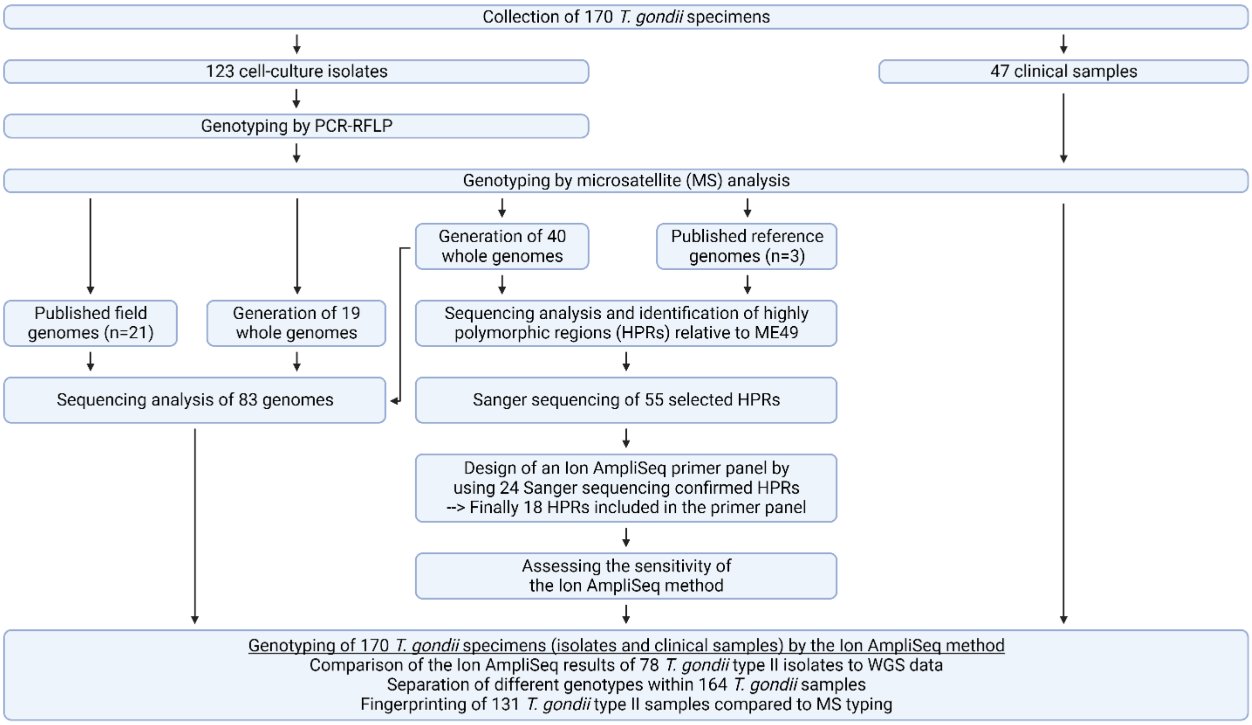

Supplementary Fig. 1 Detailed workflow of the establishment of the Ion AmpliSeq method. Created with BioRender.com

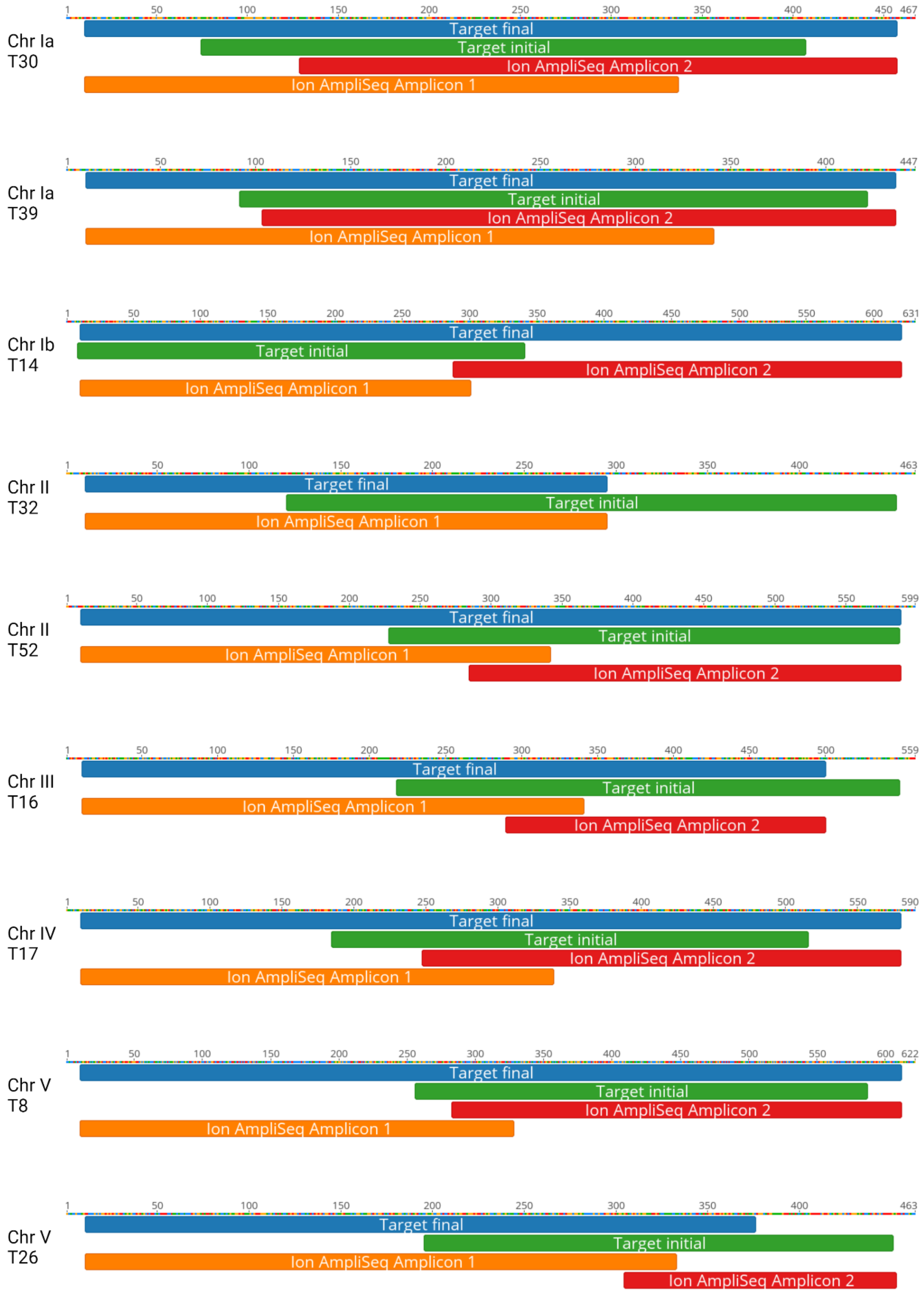

Supplementary Fig. 2a

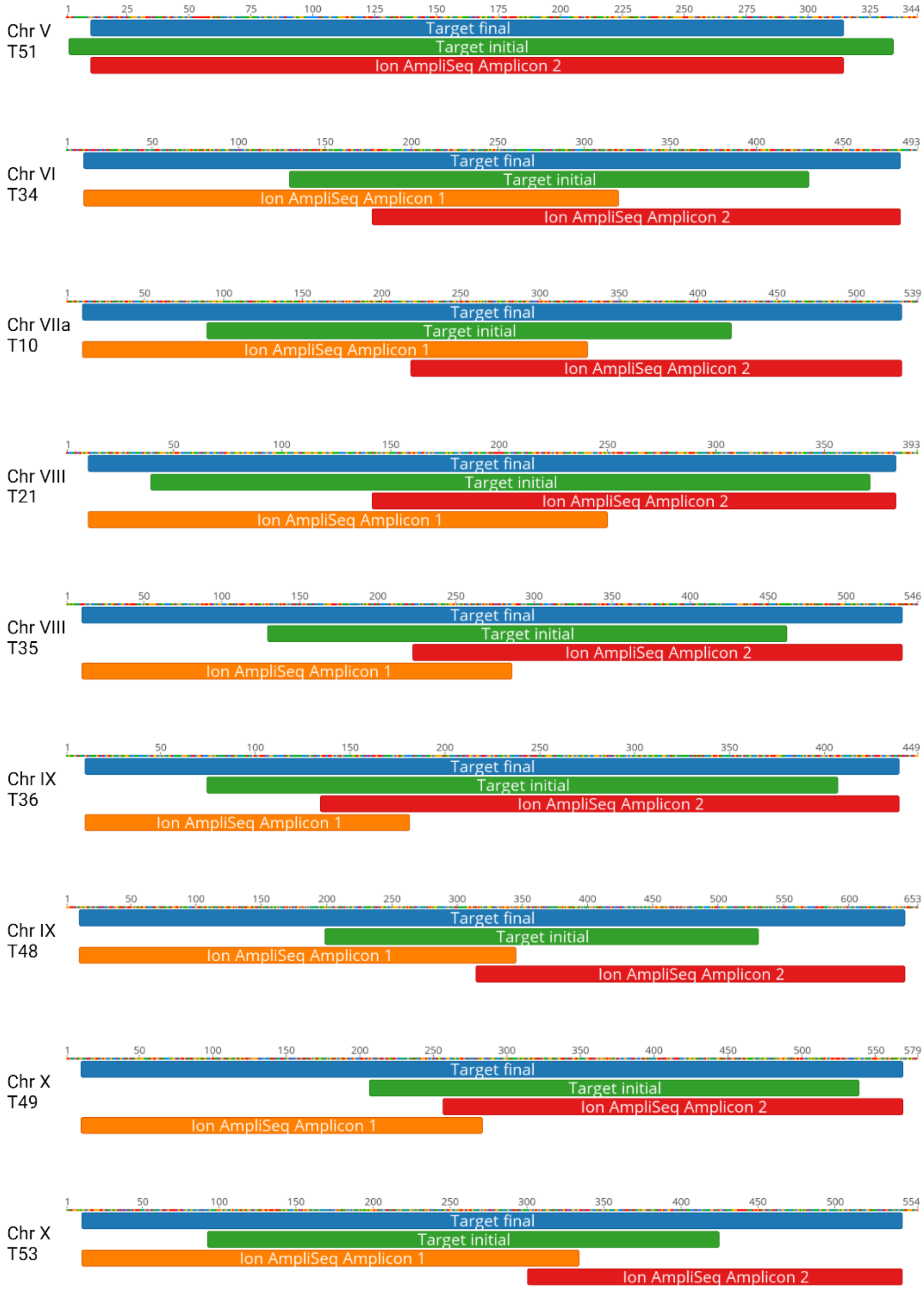

Supplementary Fig. 2b

Supplementary Fig. 2a-2b Schematic presentation of the locations of the Ion AmpliSeq target regions and Ion AmpliSeq amplicons. “Target final”, colored in blue, shows the regions used for SNP analysis and consists of the Ion AmpliSeq amplicons colored in orange and red. An exception is target region T26, which was shortened for SNP analysis because runs of consecutive thymine nucleotides (poly(T)) had led to ambiguous sequencing results. “Target initial”, colored in green, shows the regions initially identified by WGS analysis and used for the Ion AmpliSeq primer design. The sequences and base numbers shown on top of each illustration correspond to those contained in the FASTA file used for Ion AmpliSeq data analysis (assessible at https://zenodo.org/; DOI: 10.5281/zenodo.8377016; named as “AmpliSeq-ME49-Reference”)

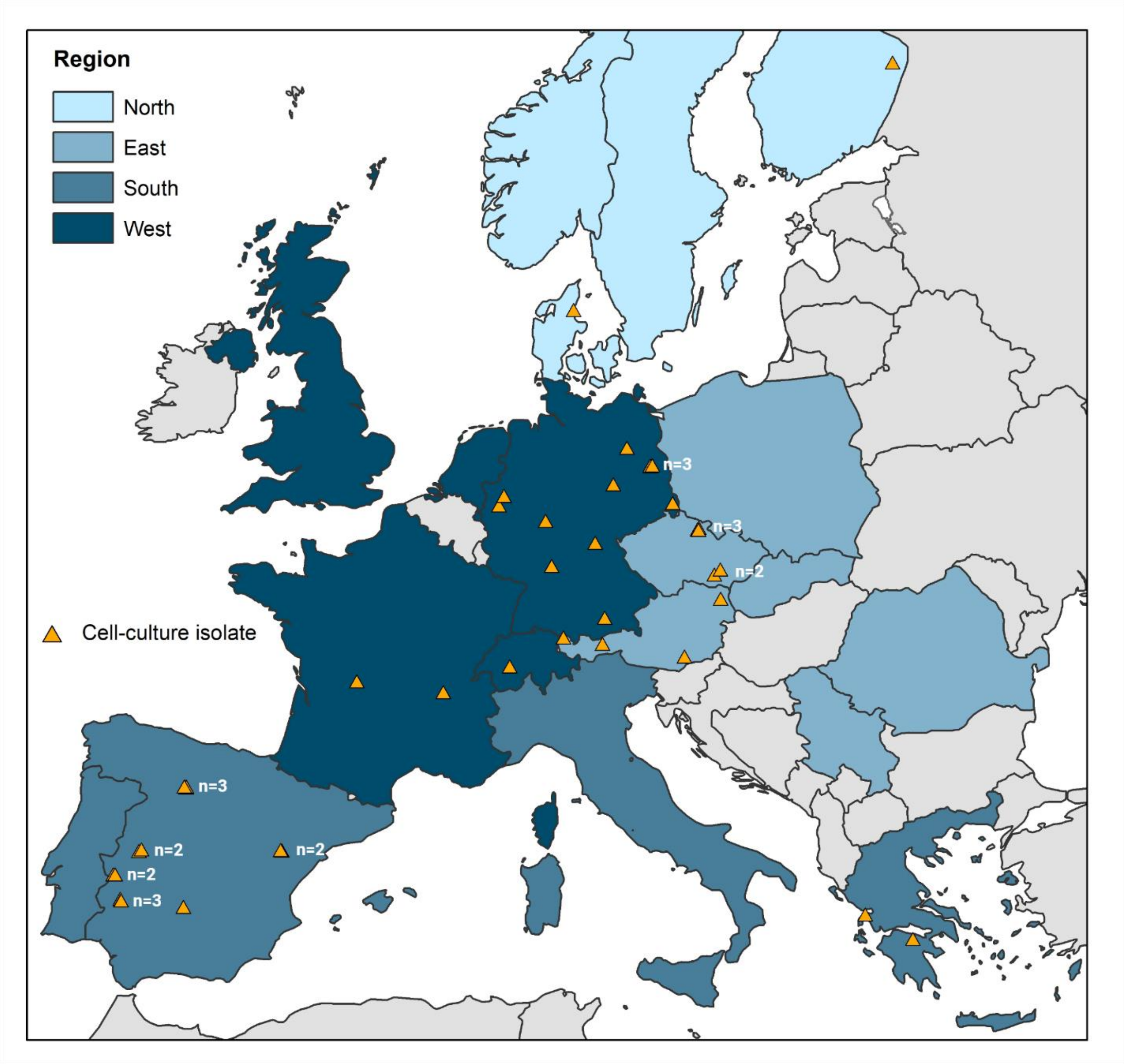

Supplementary Fig. 3 Geographic origin of 42 European isolates among those isolates (n=43) used for the identification of highly polymorphic regions relative to the Non-European ME49 genome. If more than one isolate originated from the same location, the number of isolates per locality is added

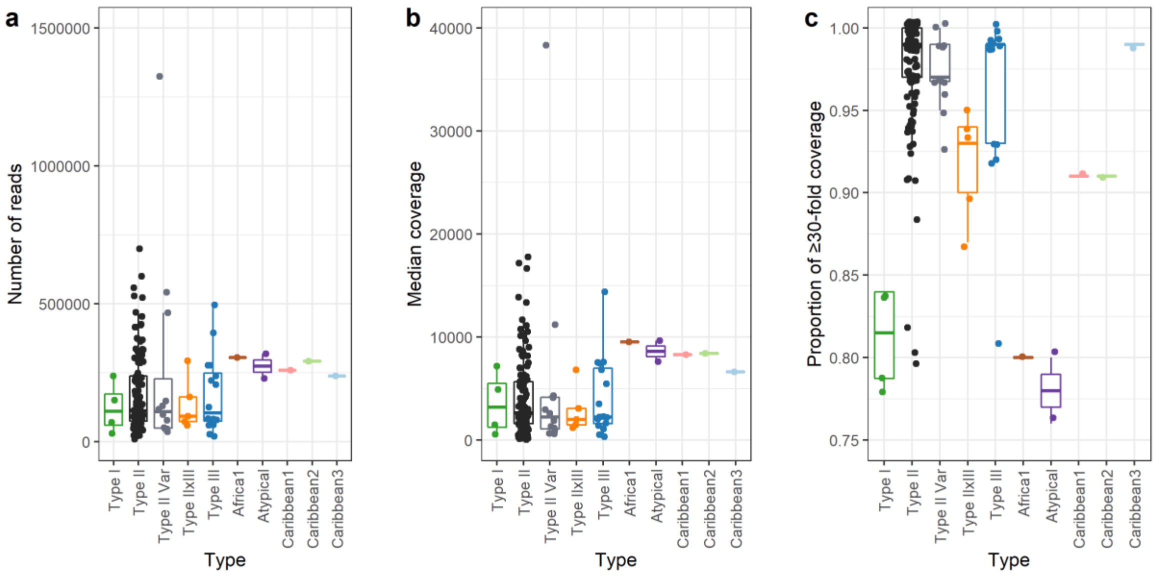

Supplementary Fig. 4 Number of reads of *T. gondii* libraries sequenced with Ion AmpliSeq sequencing and coverage of the AmpliSeq-ME49-Reference after mapping of the reads. The figure includes the results of 121 type II specimens, twelve type II variants, 14 type III (excluding C25 and C26, classified as ToxoDB #123 by PCR-RFLP typing) and four type I specimens. Only one specimen each was analyzed in case of Africa 1 and Caribbean 1, 2 and 3 and in addition two atypical specimens and five type IIxIII recombinants were examined. (a) Number of total reads per genotype. (b) Median coverage of the AmpliSeq-ME49-Reference shows a distribution per genotype that is comparable to the total number of reads. (c) Proportion of target regions with a ≥30-fold coverage revealed that some regions are not covered by the reads of the genotypes type I, Africa 1, Atypical, Caribbean 1 and Caribbean 2

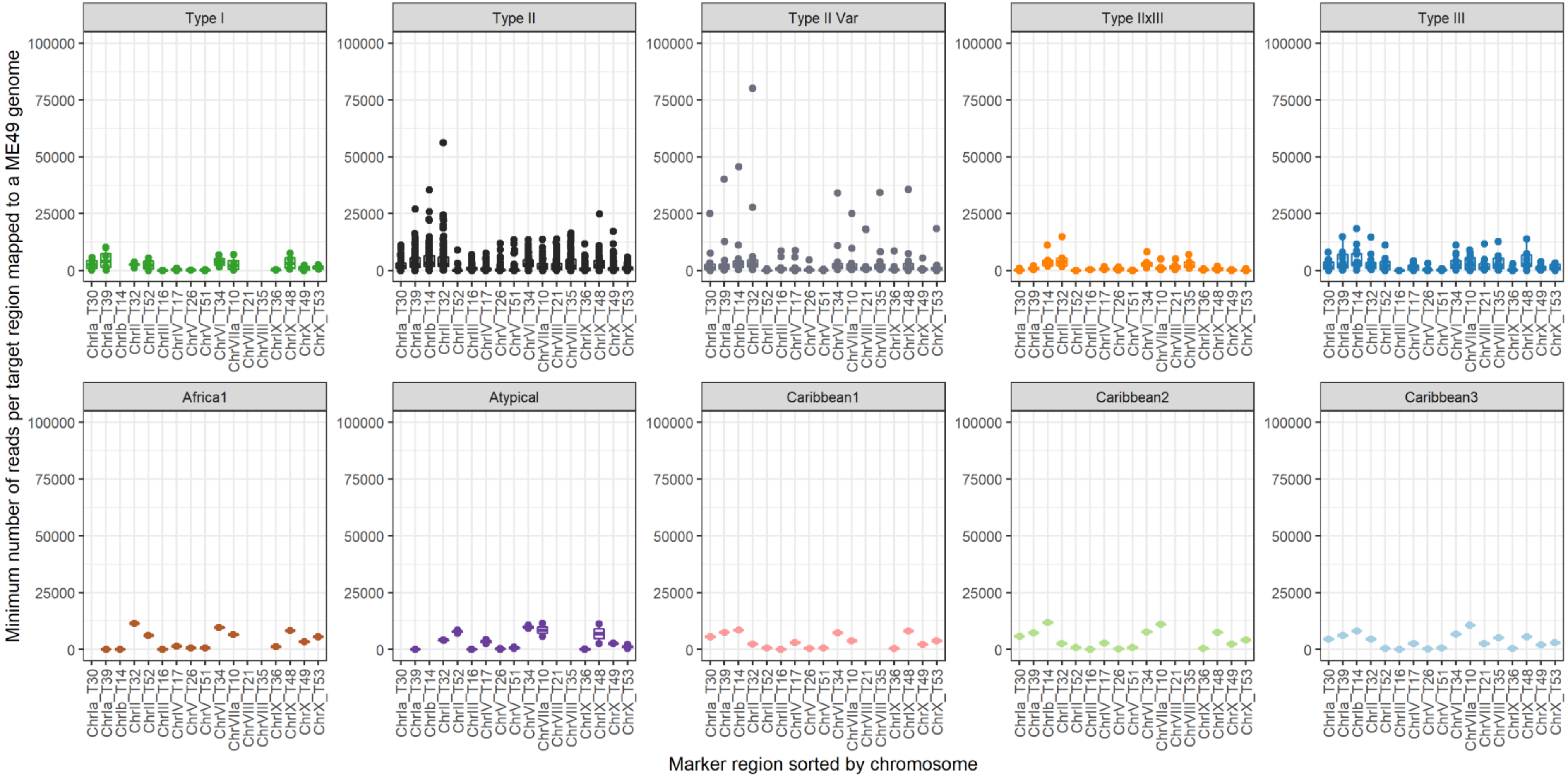

Supplementary Fig. 5a

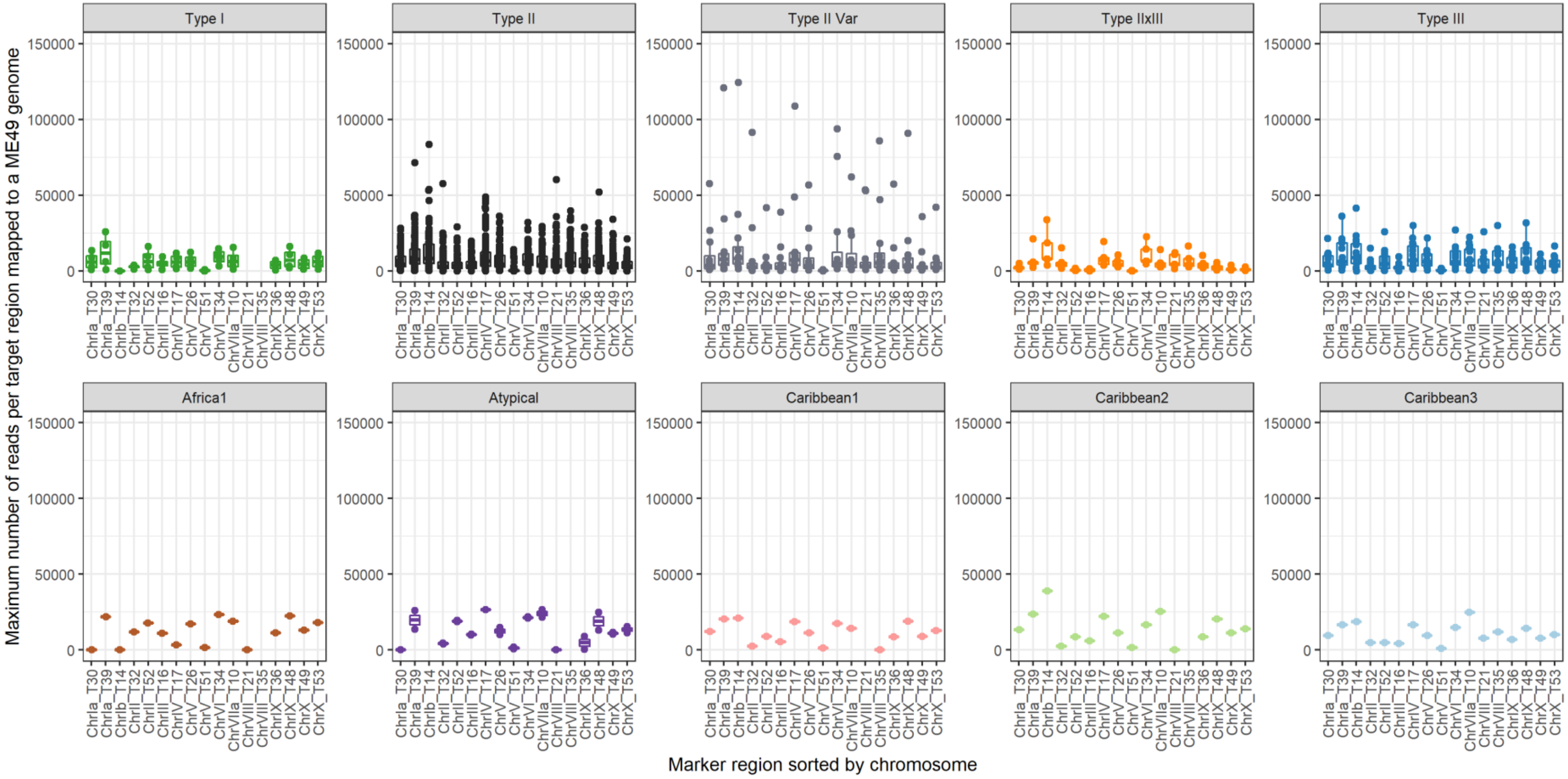

Supplementary Fig. 5b

Supplementary Fig. 5a-5b Minimum and maximum numbers of reads per target region mapped the genome of ME49 (ToxoDB release 53). The figure summarizes results for 121 type II specimens, twelve type II variants, 14 type III (excluding C25 and C26, classified as ToxoDB #123 by PCR-RFLP typing) and four type I specimens. Only one specimen each was analyzed in case of Africa 1 and Caribbean 1, 2 and 3, and in addition two atypical specimens and five type IIxIII recombinants were examined. If a boxplot is missing, the affected target region was not covered by the reads of the respective genotype. This was the case for the atypical specimens in target region T14 as well as for type I, Africa 1, Caribbean 1, Caribbean 2 and atypical specimens in the target regions T21 and T35

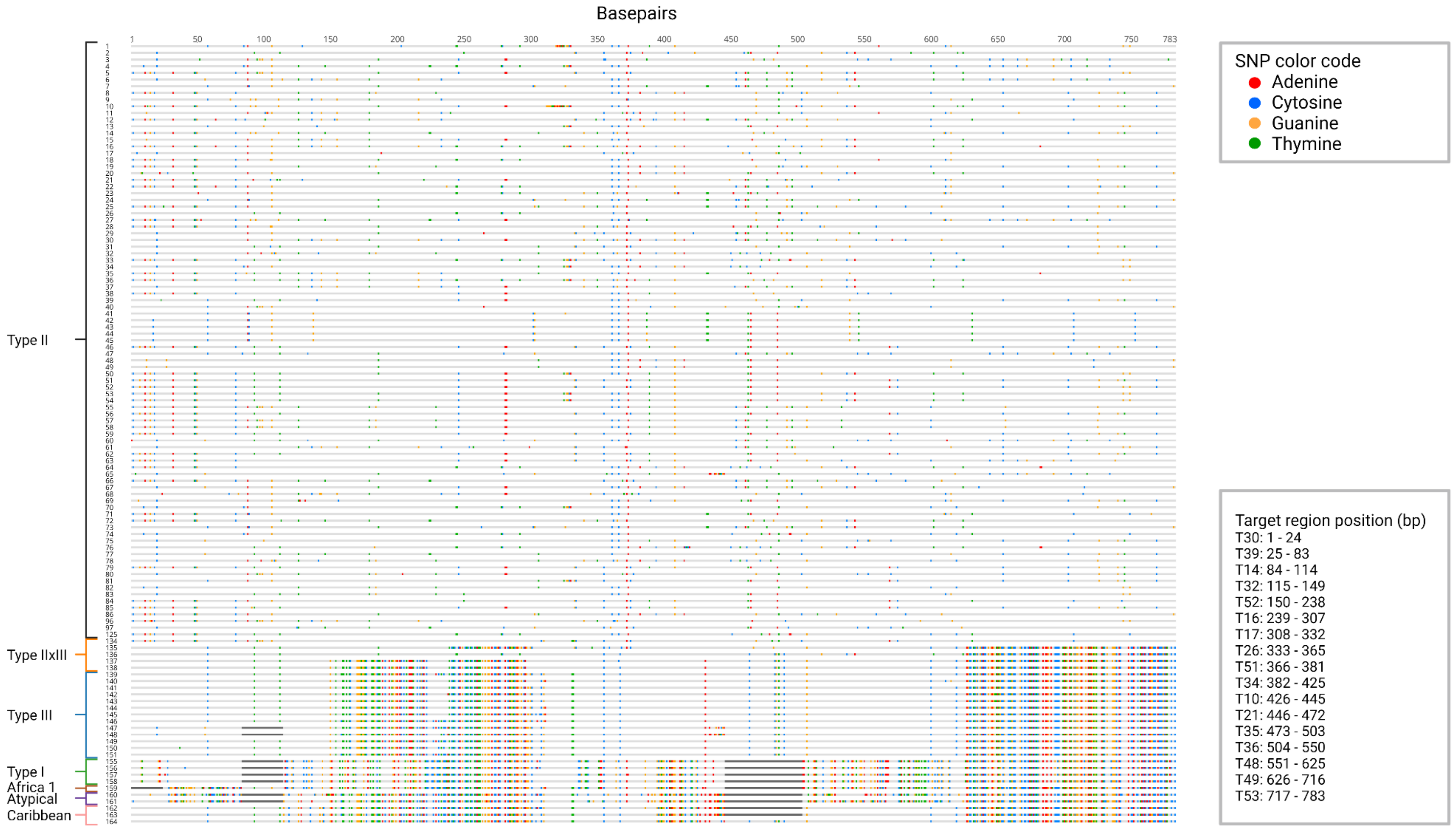

Supplementary Fig. 6 Overview of the SNP distribution of 123 *T. gondii* isolates relative to the AmpliSeq-ME49-Reference, sorted by genotype. The basis for this illustration was a FASTA file containing only those parts of the reference sequence, where SNPs had been observed. If parts of the reference were not covered by the reads of specific genotypes, the corresponding nucleotides were indicated as "N" in the FASTA file, colored dark grey in the illustration

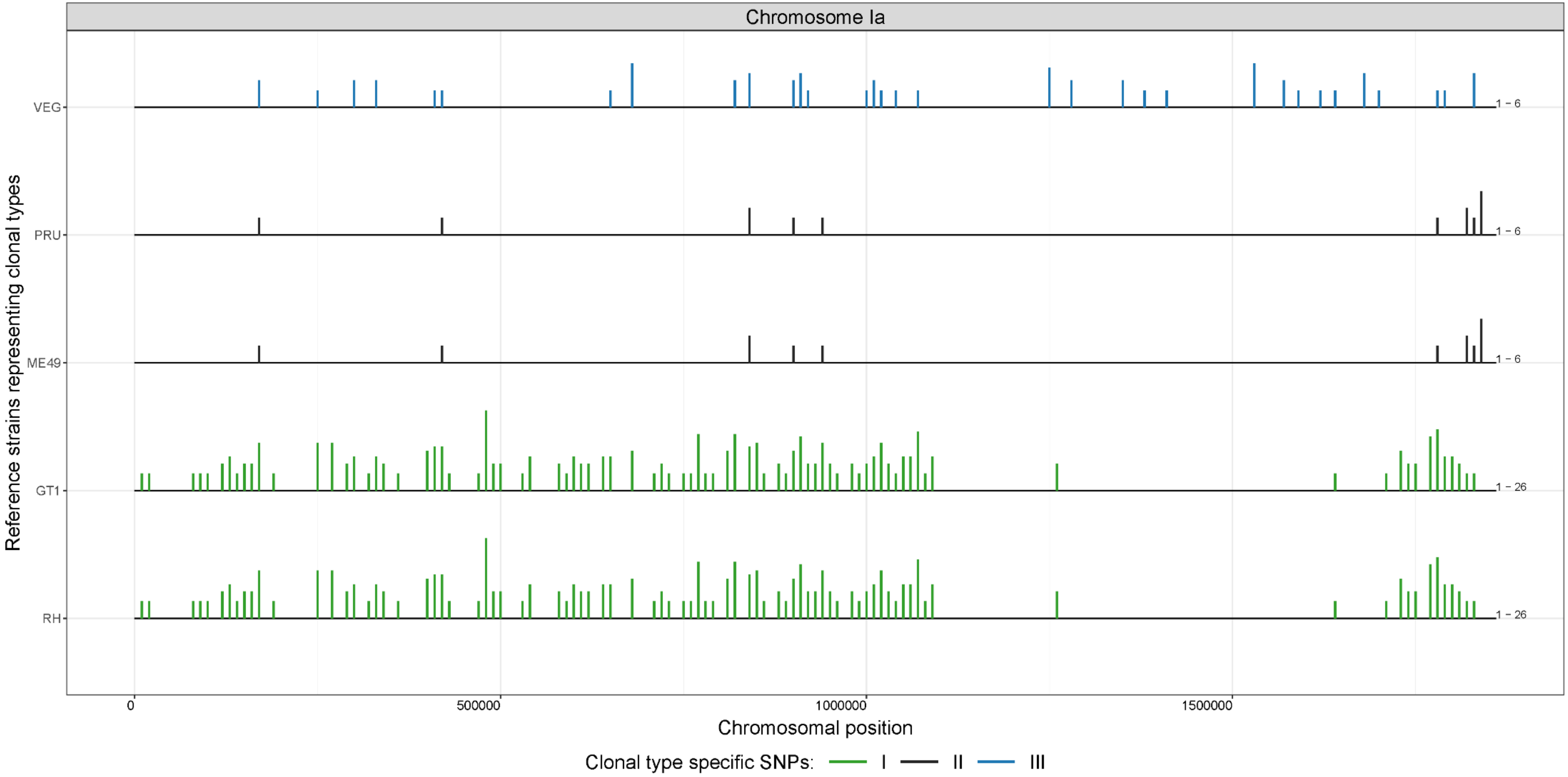

Supplementary Fig. 7a

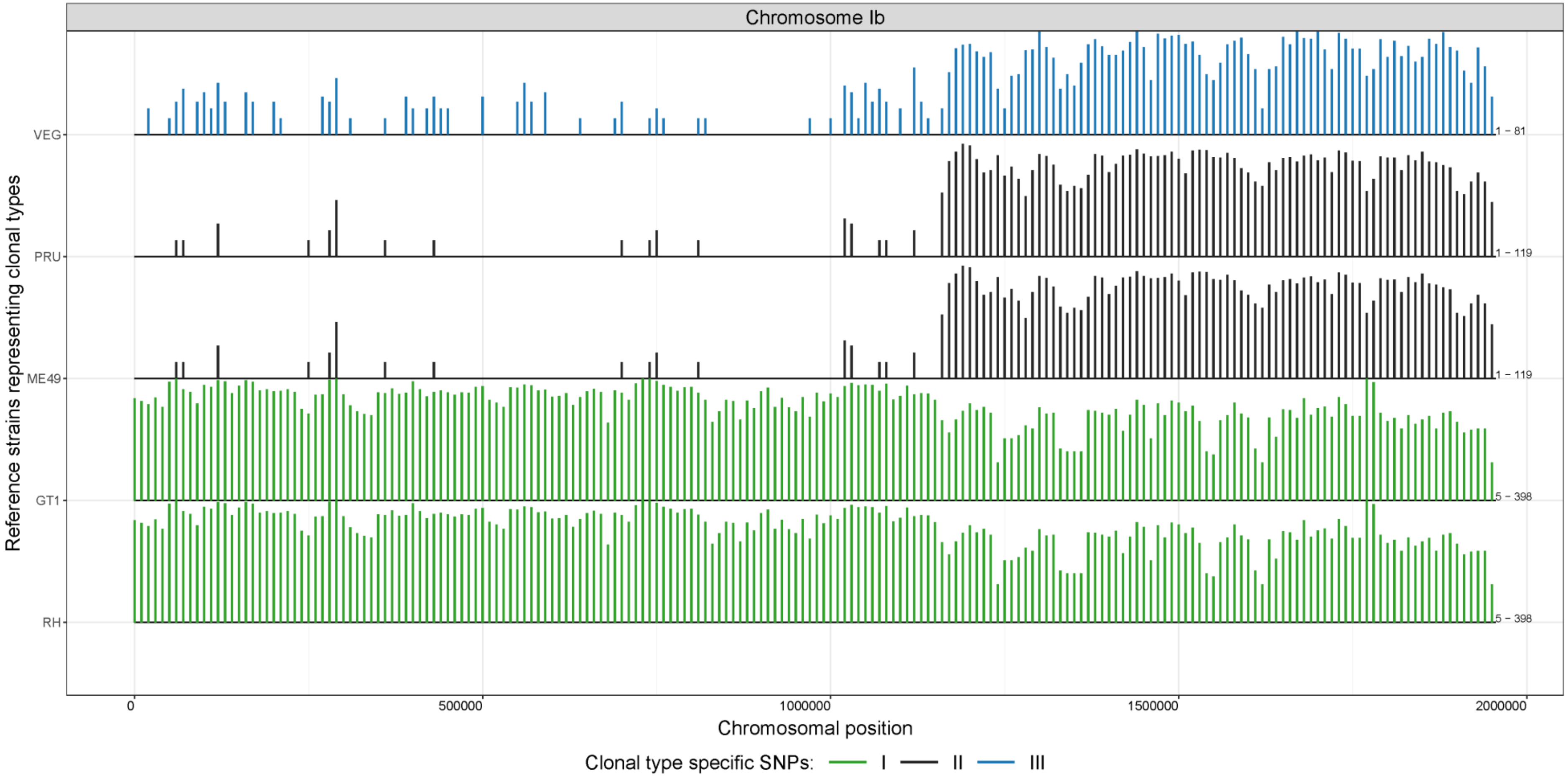

Supplementary Fig. 7b

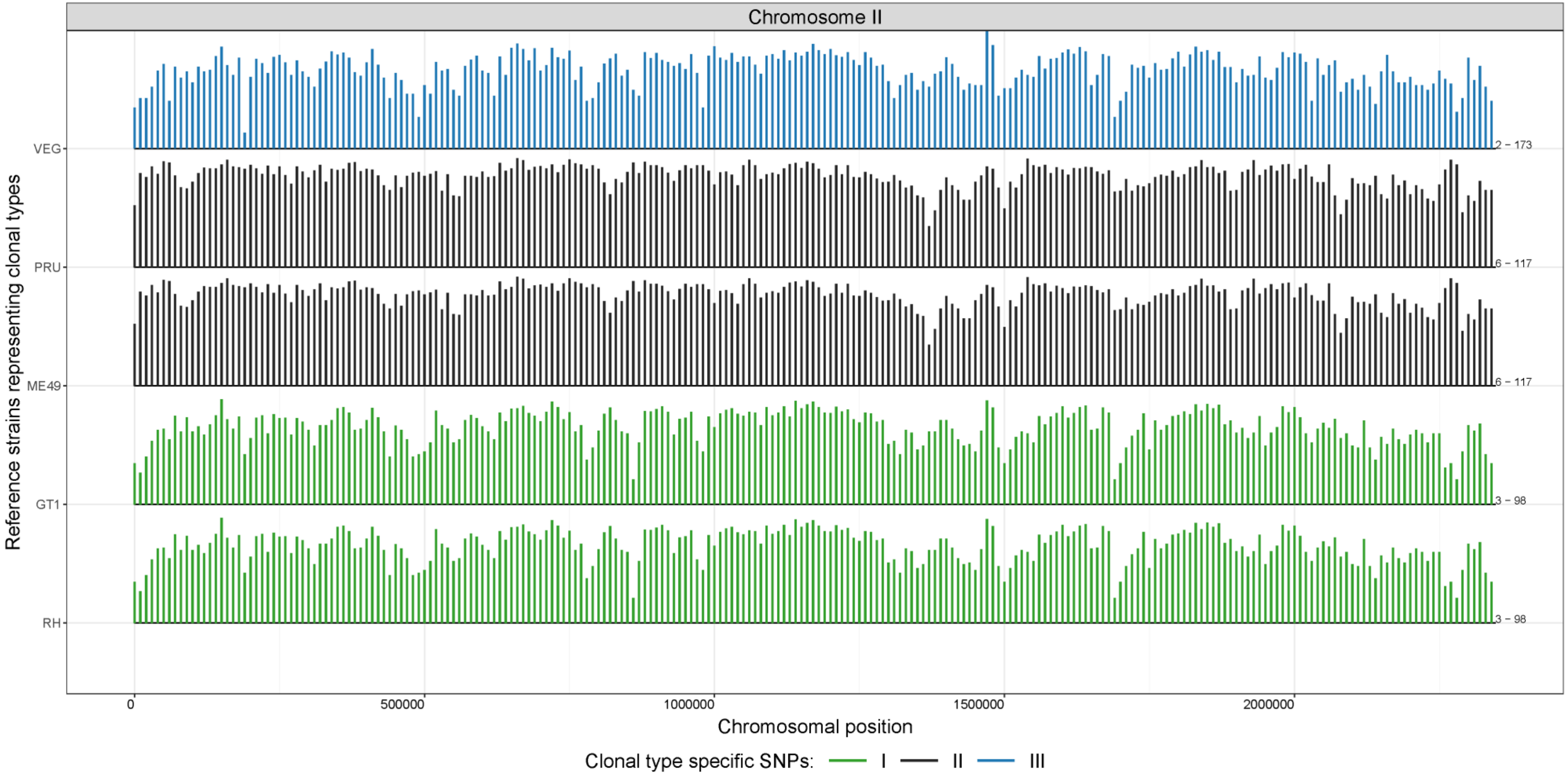

Supplementary Fig. 7c

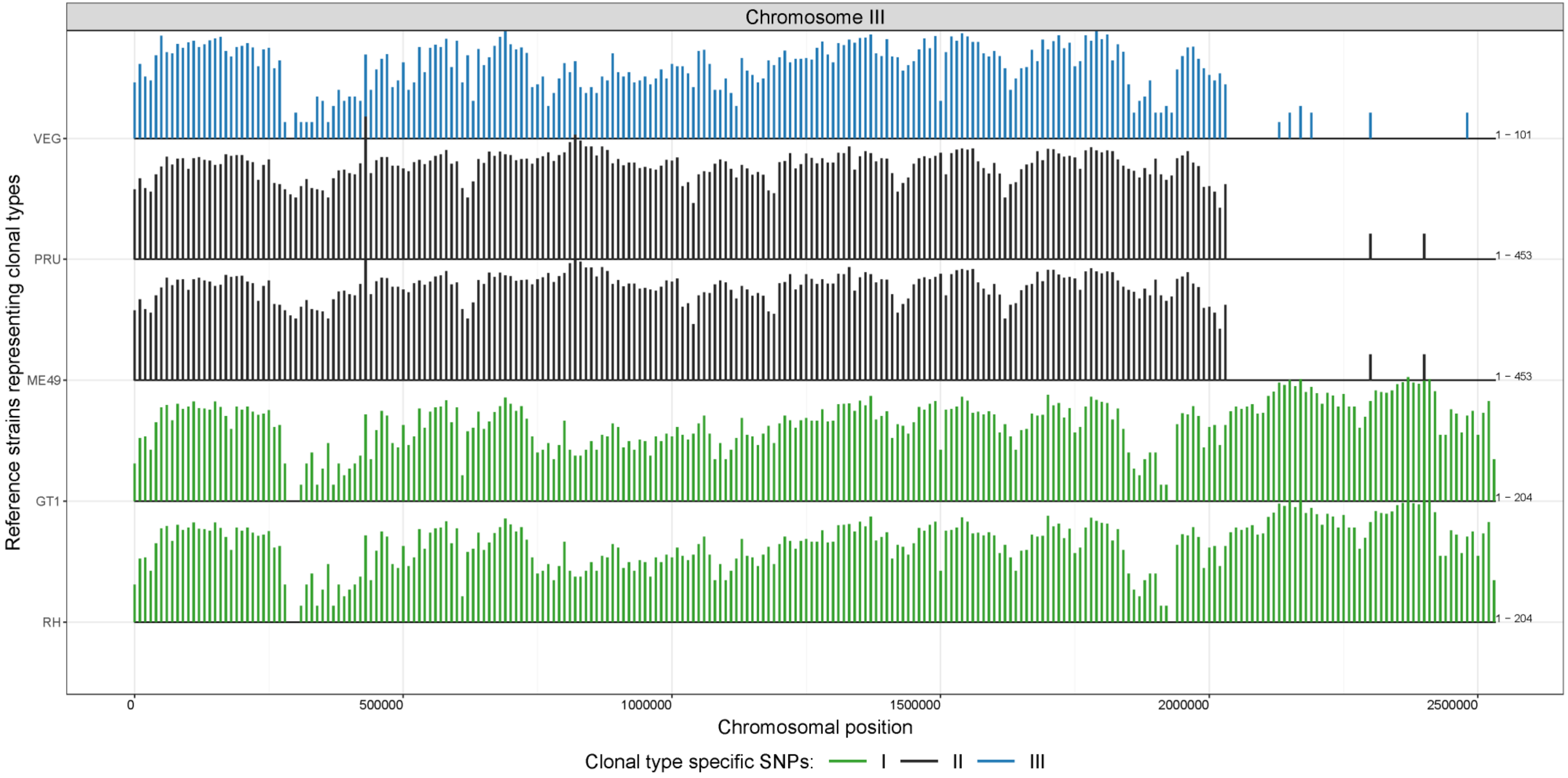

Supplementary Fig. 7d

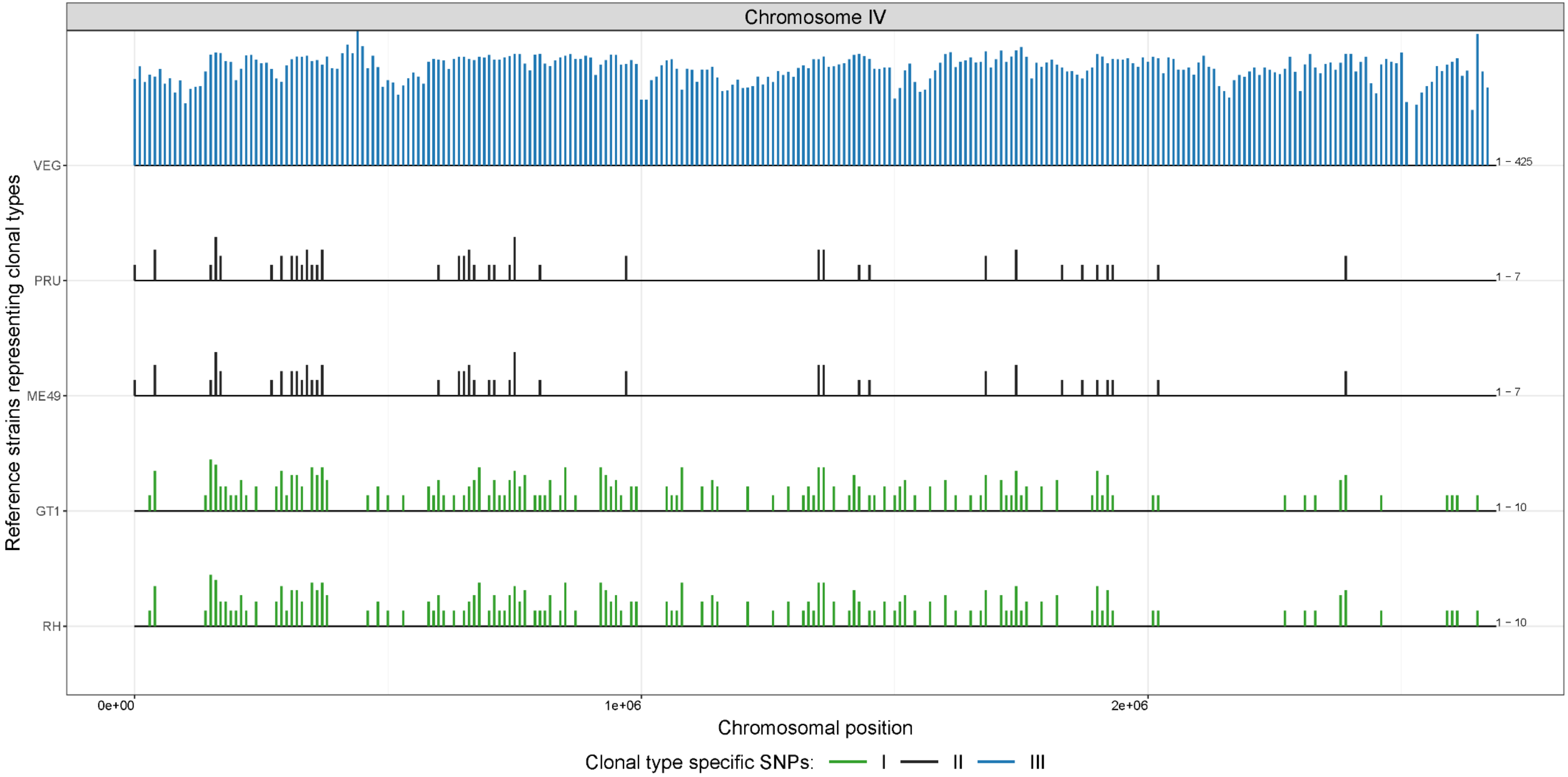

Supplementary Fig. 7e

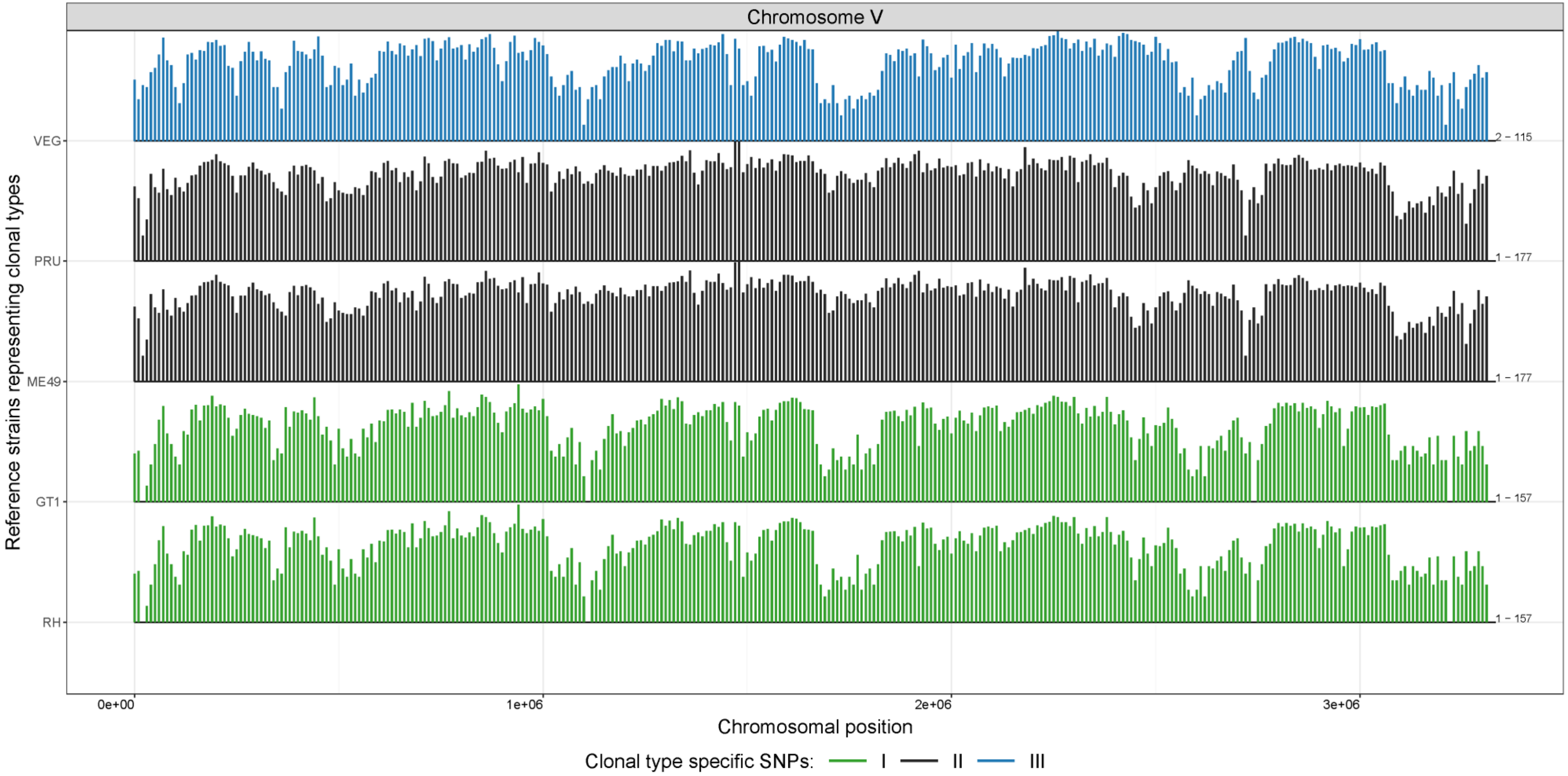

Supplementary Fig. 7f

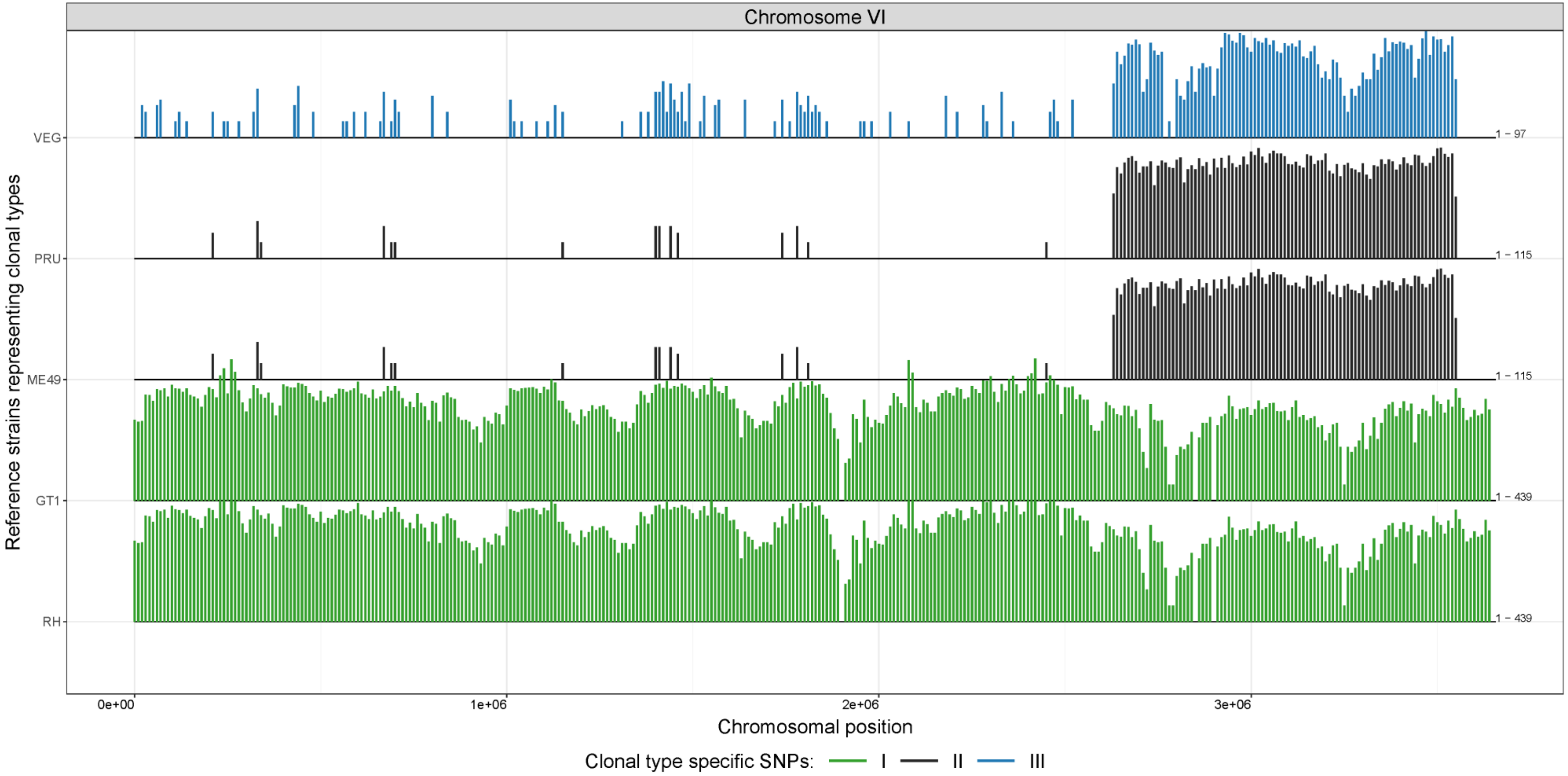

Supplementary Fig. 7g

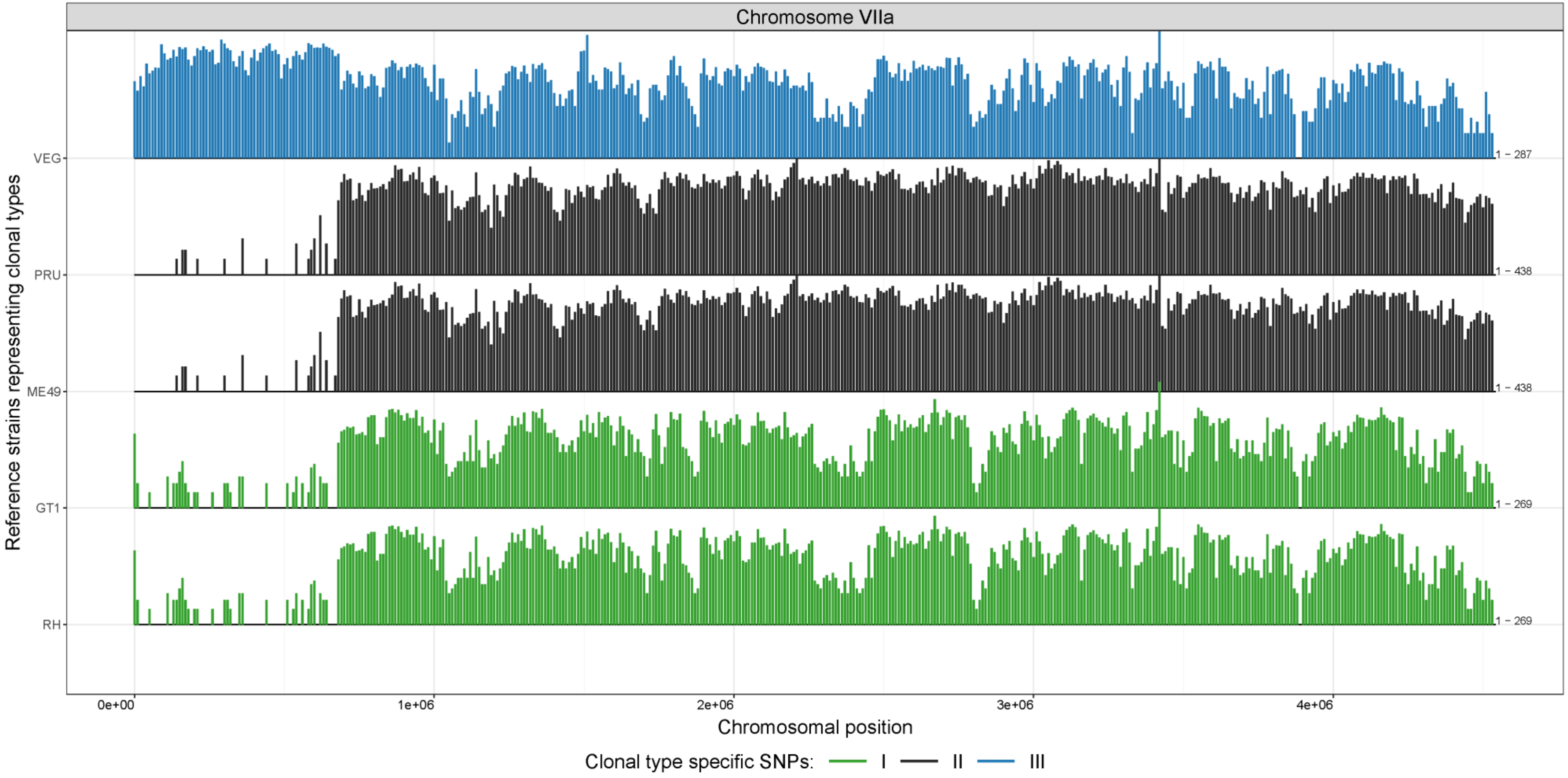

Supplementary Fig. 7h

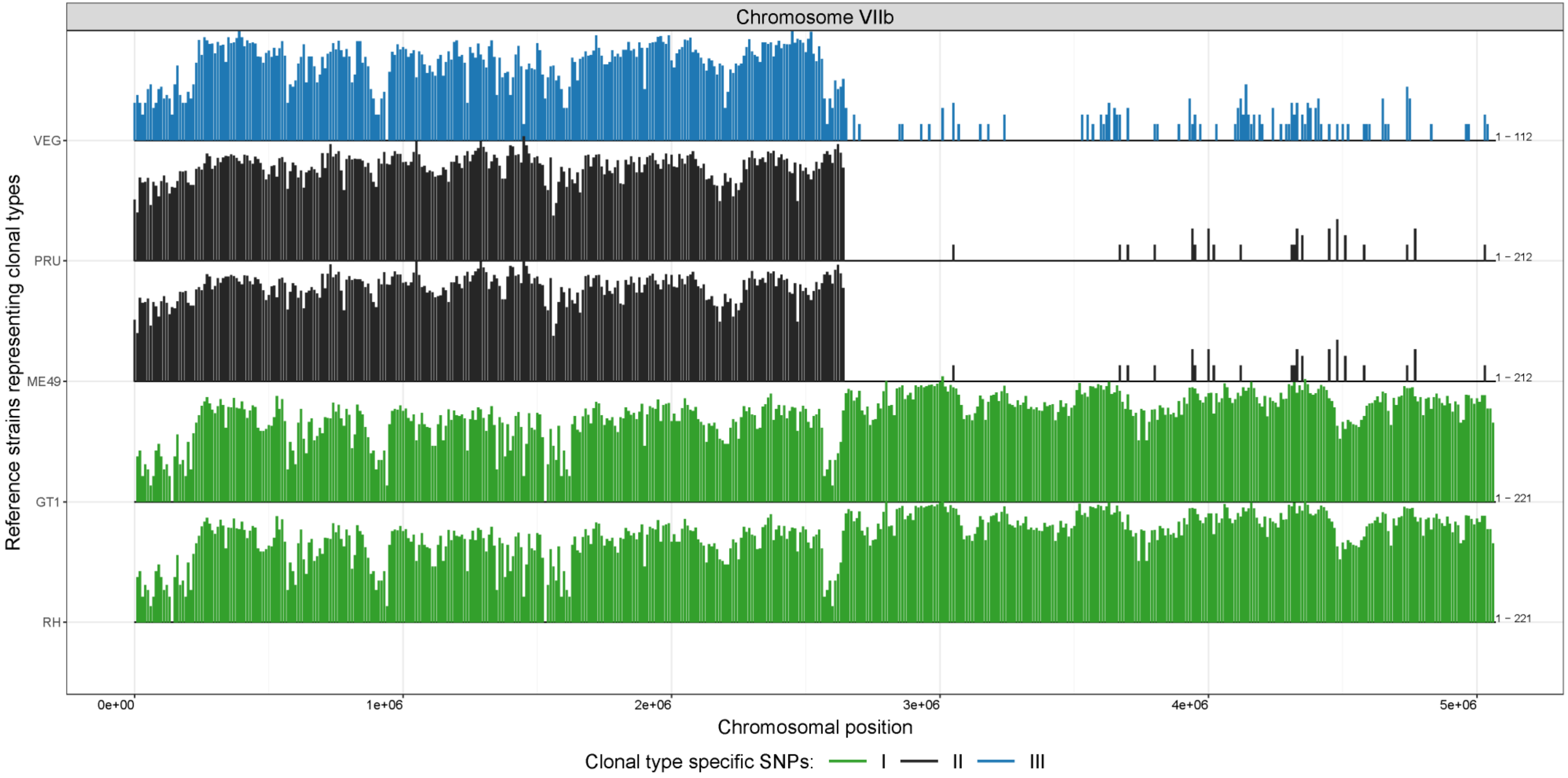

Supplementary Fig. 7i

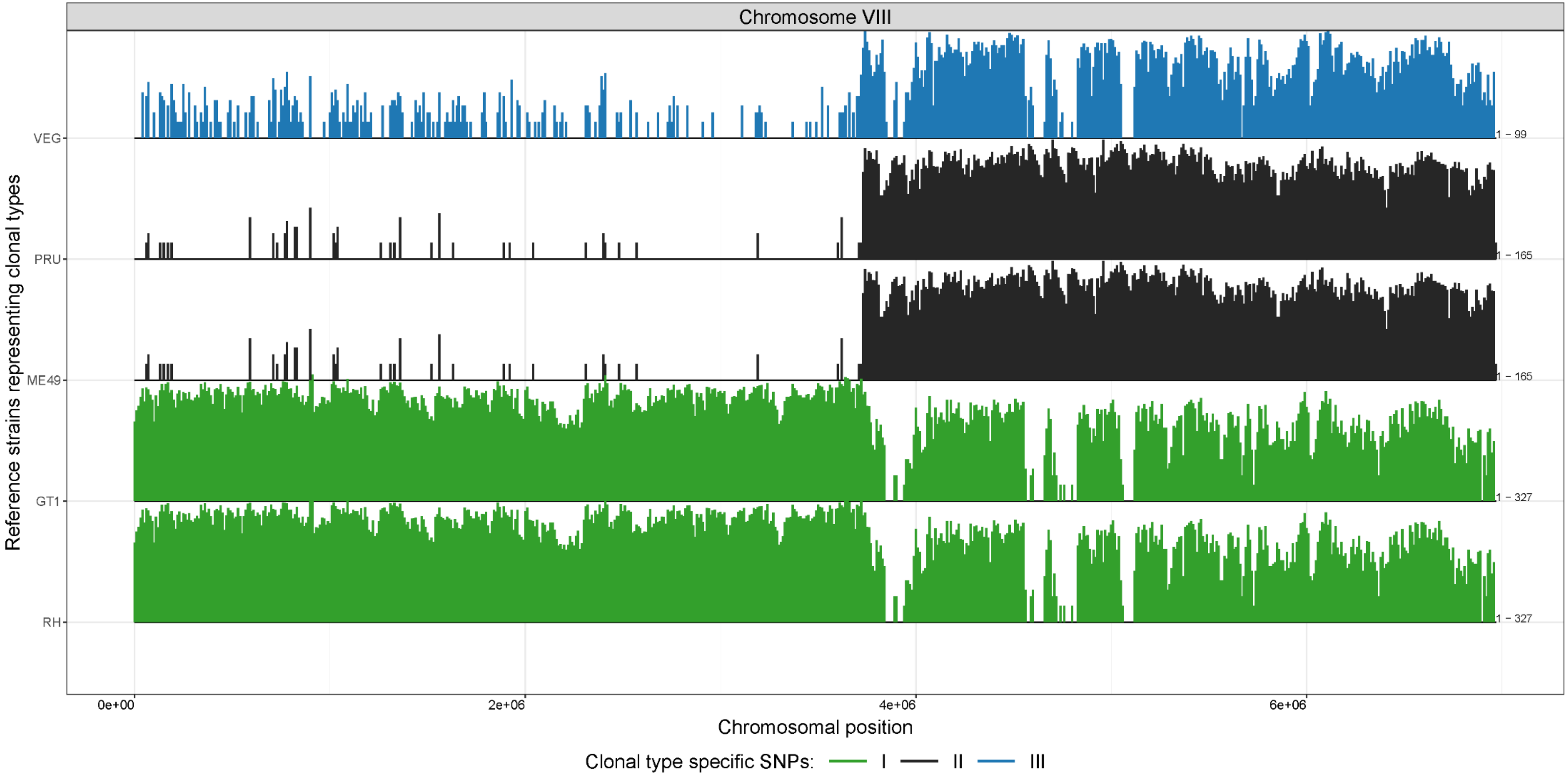

Supplementary Fig. 7j

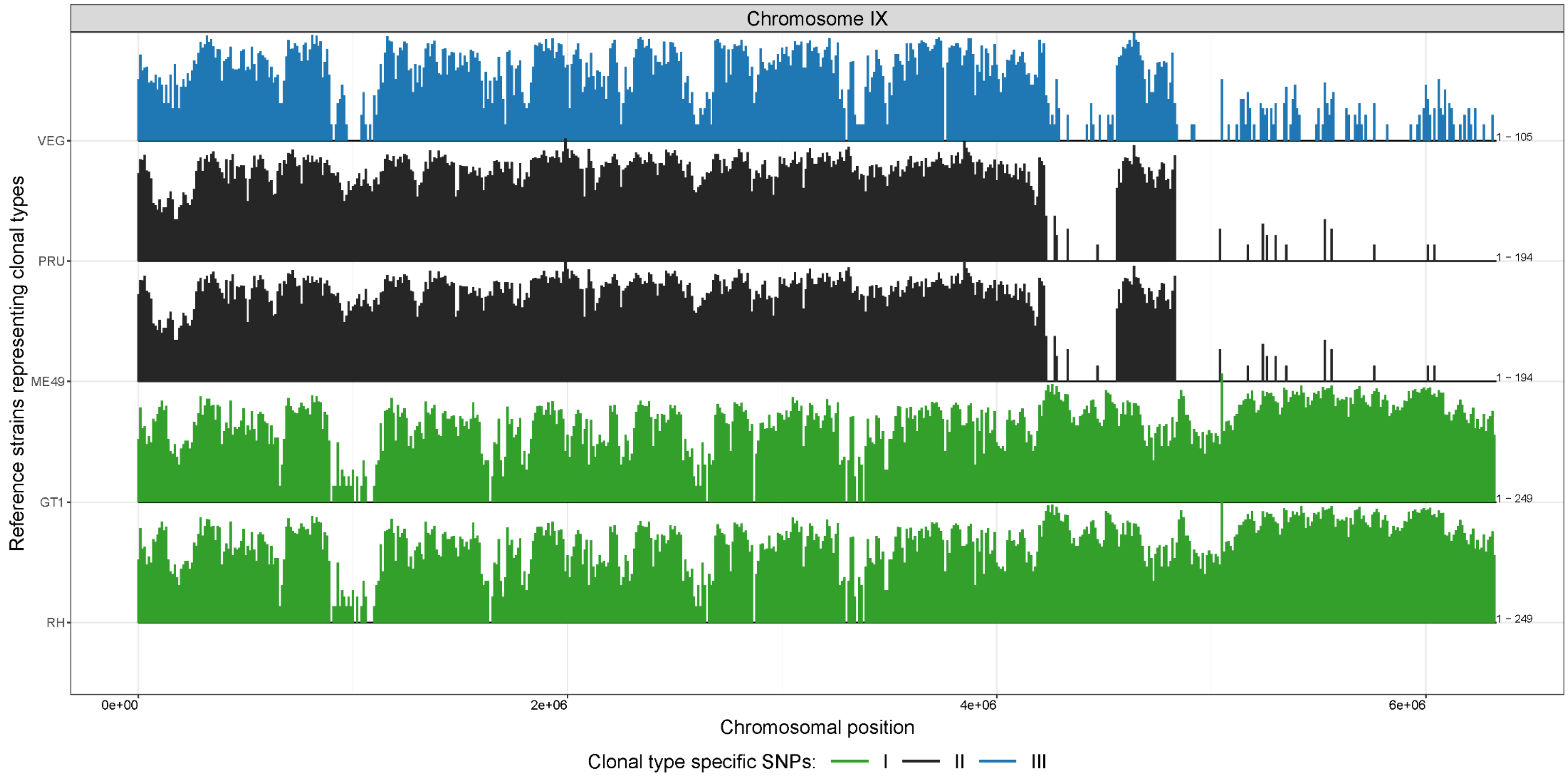

Supplementary Fig. 7k

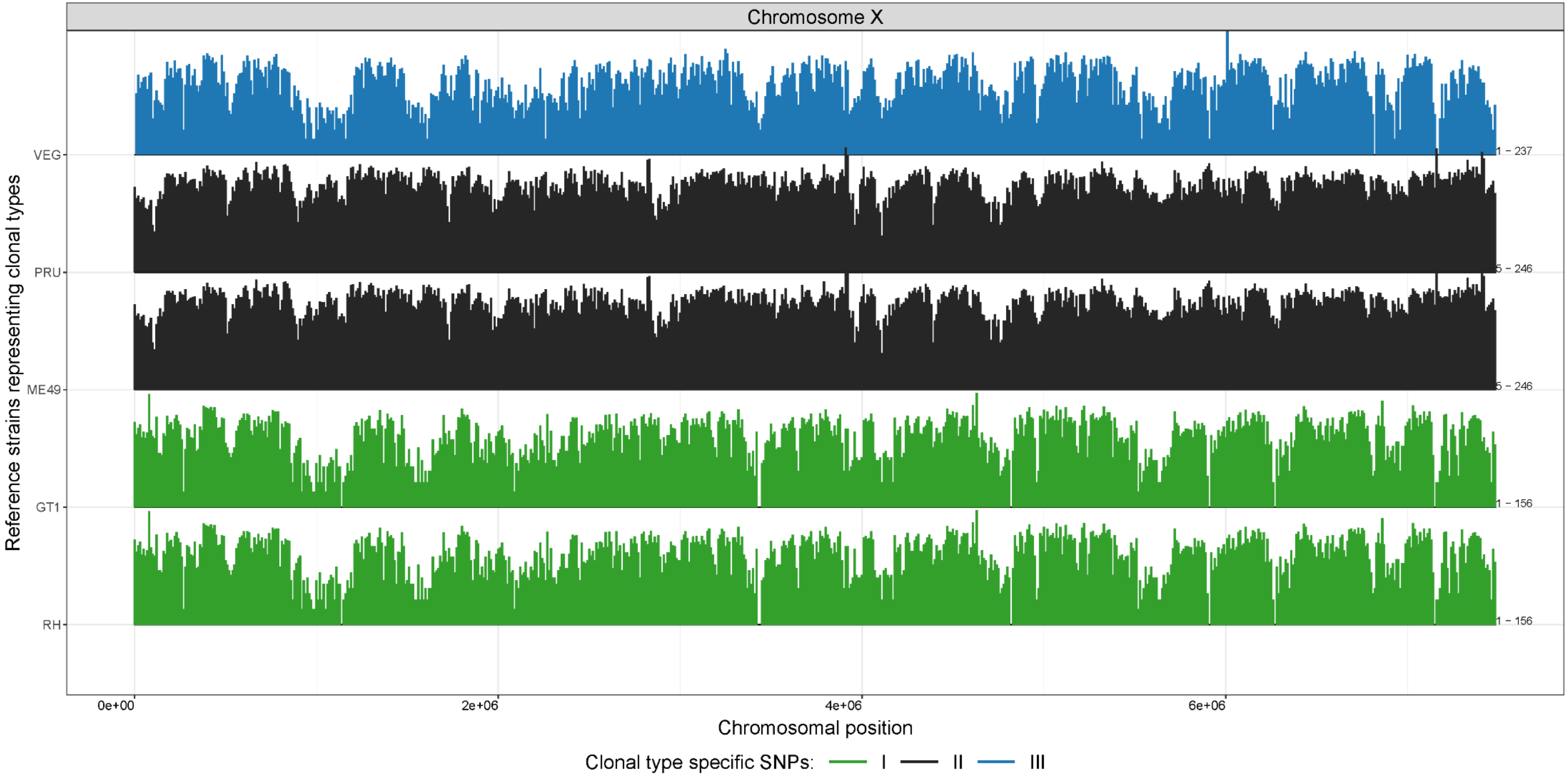

Supplementary Fig. 7l

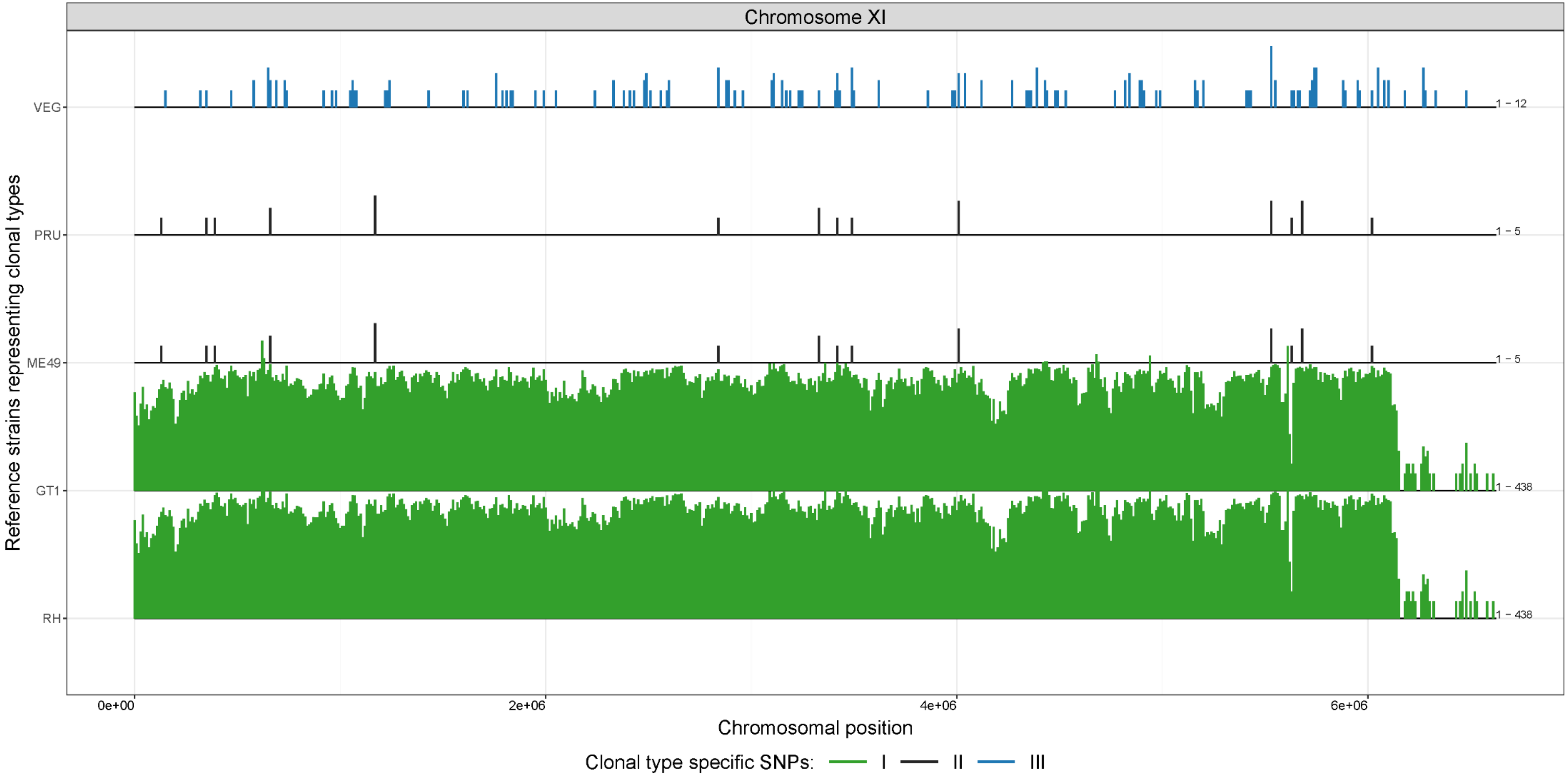

Supplementary Fig. 7m

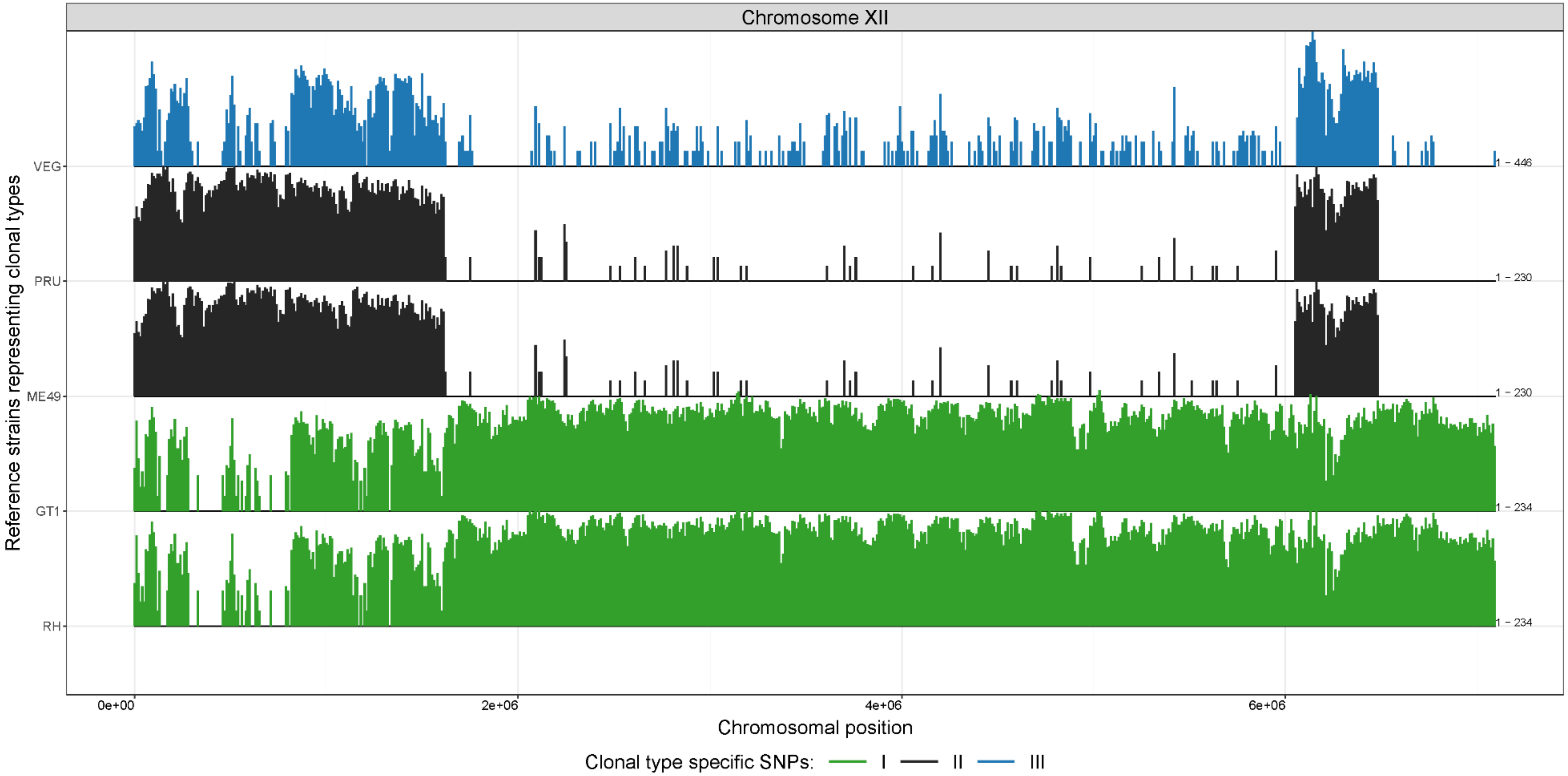

Supplementary Fig. 7n

Supplementary Fig. 7a-7n Type-specific distribution of SNPs per chromosome of *T. gondii* type I, II and III reference strains relative to the genome of ME49 (ToxoDB release 53)**.** For the annotation of type-specific SNPs, whole genome sequences for strains of *T. gondii* clonal lineages type I, II and III were downloaded from the European Nucleotide Archive (https://www.ebi.ac.uk/ena/browser/home). Sequences of two *T. gondii* type I strains (RH (SRR521559) and GT1 (SRR516419)), two type II strains (ME49 (SRR67938639 and PRU (SRR350739)) and one type III strain (VEG (ERR701180)) were mapped against the whole genome reference sequence of ME49 (ToxoDB release 53). SNPs were called and filtered from the mapped reads using GATK tools as previously described in Supplementary Note 2. Subsequently, the SNPs identified among the three clonal lineages, were defined as type I, II, or III specific; i.e. a SNP was regarded as specific for one of the types if it revealed to be polymorphic relative to the remaining types. If two strains per lineage were used, only type-specific SNPs present in both strains (PRU and ME49 or RH and GT1) were considered. Each bar indicates the number of SNPs per 10 kb windows of the chromosomes and minimum and maximum number of SNPs per 10 kb are given on the right side for each chromosome and strain

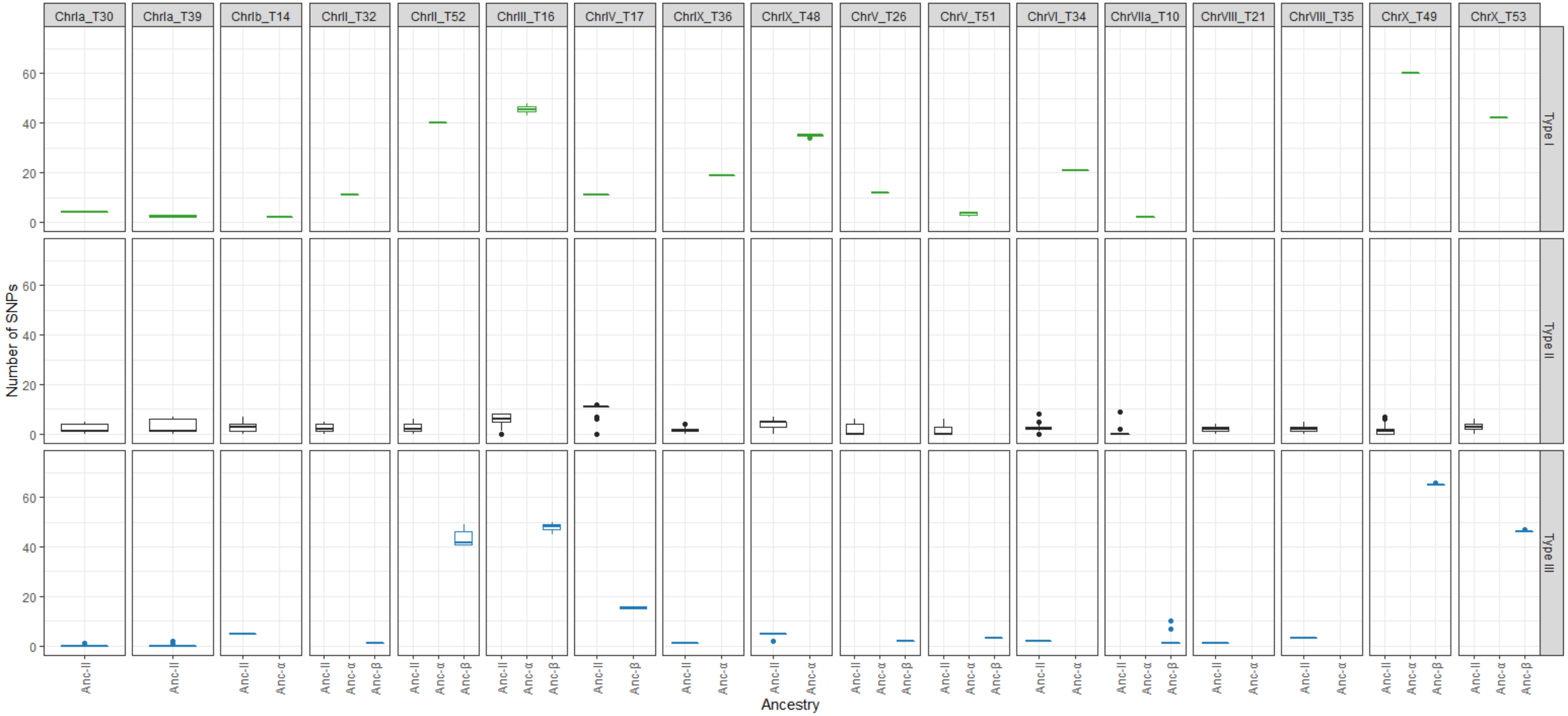

Supplementary Fig. 8 Numbers of SNPs detected by Ion AmpliSeq typing within four type I, 121 type II and 14 type III specimens relative to AmpliSeq-ME49-Reference combined with the genealogy of these *T. gondii* lineages as proposed by Boyle et al. (2006). Boyle et al. (2006) suggested the chromosomes of type I and type III being a cross of Ancestral type II (Anc-II) x Ancestral α (Anc-α) and Ancestral type II x Ancestral β (Anc-β), respectively. The shown regions and the associated SNPs were differentiated into Ancestral type II (Anc-II) and Ancestral α (Anc-α) and Ancestral β (Anc-β), respectively, based on Suppl. Fig. 7

Reference: Boyle JP, Rajasekar B, Saeij JPJ et al. (2006) Just one cross appears capable of dramatically altering the population biology of a eukaryotic pathogen like *Toxoplasma gondii*. Proc Natl Acad Sci U S A 103:10514–10519. https://doi.org/10.1073/pnas.0510319103

Funding

This work was part of TOXOSOURCES project, supported by funding from the European Union's Horizon 2020 Research and Innovation programme under grant agreement No 773830: One Health European Joint Programme.
